# A novel SfaNI-like restriction-modification system in *Caldicellulosiruptor* extents the genetic engineering toolbox for this genus

**DOI:** 10.1371/journal.pone.0279562

**Published:** 2022-12-29

**Authors:** Steve Swinnen, Christian Zurek, Marco Krämer, Rebecca M. Heger, Jan-Eike Domeyer, Jan Ziegler, Vitali A. Svetlitchnyi, Albrecht Läufer

**Affiliations:** 1 BluCon Biotech GmbH, Cologne, Germany; 2 BRAIN Biotech AG, Zwingenberg, Germany; University of Illinois at Urbana-Champaign, UNITED STATES

## Abstract

*Caldicellulosiruptor* is a genus of thermophilic to hyper-thermophilic microorganisms that express and secrete an arsenal of enzymes degrading lignocellulosic biomasses into fermentable sugars. Because of this distinguished feature, strains of *Caldicellulosiruptor* have been considered as promising candidates for consolidated bioprocessing. Although a few *Caldicellulosiruptor* strains with industrially relevant characteristics have been isolated to date, it is apparent that further improvement of the strains is essential for industrial application. The earlier identification of the HaeIII-like restriction-modification system in *C*. *bescii* strain DSM 6725 has formed the basis for genetic methods with the aim to improve the strain’s lignocellulolytic activity and ethanol production. In this study, a novel SfaNI-like restriction-modification system was identified in *Caldicellulosiruptor* sp. strain BluCon085, consisting of an endonuclease and two methyltransferases that recognize the reverse-complement sequences 5’-GATGC-3’ and 5‘-GCATC-3’. Methylation of the adenine in both sequences leads to an asymmetric methylation pattern in the genomic DNA of strain BluCon085. Proteins with high percentage of identity to the endonuclease and two methyltransferases were identified in the genomes of *C*. *saccharolyticus* strain DSM 8903, *C*. *naganoensis* strain DSM 8991, *C*. *changbaiensis* strain DSM 26941 and *Caldicellulosiruptor* sp. strain F32, suggesting that a similar restriction-modification system may be active also in these strains and respective species. We show that methylation of plasmid and linear DNA by the identified methyltransferases, obtained by heterologous expression in *Escherichia coli*, is sufficient for successful transformation of *Caldicellulosiruptor* sp. strain DIB 104C. The genetic engineering toolbox developed in this study forms the basis for rational strain improvement of strain BluCon085, a derivative from strain DIB 104C with exceptionally high L-lactic acid production. The toolbox may also work for other species of the genus *Caldicellulosiruptor* that have so far not been genetically tractable.

## Introduction

The emergence of climate change and environmental pollution has been an important driver for the advancement of a biobased chemicals industry that relies on biocatalysts to convert renewable materials into value-added products [1, 2]. Based on the type of renewable materials, biochemicals are generally designated as first- or second-generation [3]. First-generation biochemicals are produced from food and feed biomasses that consist of easily accessible glucose or starch. Because of the high price of food and feed biomasses and the ethical dilemma regarding their exploitation for the production of biochemicals, focus has been laid on the use of waste biomasses and energy crops for the production of second-generation biochemicals. Waste biomasses and energy crops are a source of lignocellulose, which consists of cellulose that is tightly embedded in a hemicellulose-lignin matrix. The liberation of fermentable sugars from lignocellulose has proven to be challenging and generally requires a harsh physical, chemical or physicochemical pretreatment step followed by an enzymatic hydrolysis step [4, 5]. The realization of competitive second-generation biochemicals strongly relies on the development of processes that maximize the liberation of fermentable sugars and minimize the generation of inhibitory by-products, while keeping capital and operating expenditures as low as possible [6, 7].

Microorganisms belonging to the genus *Caldicellulosiruptor* have been considered before as interesting biocatalysts for the production of biochemicals from lignocellulosic biomasses [8–11]. Their value lies in the fact that they express and secrete a broad arsenal of enzymes that can degrade untreated lignocellulosic biomasses into fermentable sugars [12, 13]. This makes them favourable microorganisms for consolidated bioprocessing (CBP), in which enzyme production, biomass hydrolysis, and fermentation are accomplished in a single process step. CBP has been described as one of the most cost-effective processes for the production of biochemicals from lignocellulosic biomasses [14].

Lee *et al*. [15] have summed up 14 species of *Caldicellulosiruptor* that have been identified over the last three decades, most of which were isolated from either hot springs, pond sediments or compost. The exact number of species is, however, not settled because some species have recently been shown to be conspecific [16]. Cells of *Caldicellulosiruptor* grow only under strict anaerobic and thermophilic to hyper-thermophilic conditions, with optimal growth temperatures from 65 to 85°C. The main fermentation products are carbon dioxide, hydrogen, acetate, ethanol and lactic acid [17, 18].

All species of *Caldicellulosiruptor* express lignocellulolytic enzymes, however, not all show the same efficiency of cellulose and lignocellulose degradation. Among the most efficient species are *C*. *bescii*, *C*. *kronotskyensis*, *C*. *danielii*, *C*. *morganii* and *C*. *naganoensis* [19–21]. The availability of whole-genome nucleotide sequences of several *Caldicellulosiruptor* species has given first insights into the correlation between a species’ lignocellulolytic activity and the occurrence of genes in its genome encoding specific lignocellulolytic enzymes [19–22].

A crucial asset for the biobased chemicals industry is the availability of biocatalysts that operate under industrially relevant conditions. Unfortunately, natural isolates of microorganisms seldom contain all favourable characteristics to become industrial production strains, and therefore strategies to engineer these characteristics into the strains have been applied. The strategies can be divided roughly into three categories: *(i)* strategies that rely on random approaches such as breeding, evolutionary adaptation, and classic mutagenesis [23–25], *(ii)* strategies that rely on rational approaches such as metabolic modelling and reverse engineering [26, 27], and *(iii)* strategies that rely on a combination of both [28]. Each strategy has its advantages and disadvantages, and the choice for a particular strategy depends on the microorganism and the characteristic to be improved. For example, random approaches are generally better equipped to solve complex characteristics, however, they are often long and tedious and have an unpredictable outcome. Rational approaches, on the other hand, are more meticulous and focused, however, they depend on the availability of relevant knowledge about the characteristic to be improved. Rational approaches also count on the presence of a genetic engineering toolbox for the microorganism of interest.

A genetic engineering toolbox must include an efficient transformation protocol for the transfer of heterologous DNA through the cell wall and membrane and the stable maintenance of the DNA inside of the cell. In many bacteria, the maintenance of heterologous DNA inside of the cell is problematic because of the occurrence of restriction-modification (R-M) systems that serve as inherent defence strategies against the invasion of DNA (*in vivo*, primarily from phages) (reviewed by Vasu and Nagaraja [29]). R-M systems in bacteria contain at least two enzymatic activities: a restriction endonuclease that recognizes and cleaves DNA at a specific sequence, and a methyltransferase that transfers a methyl group to the same specific sequence. As methylation by a methyltransferase protects DNA from cleavage by the related restriction endonuclease, the cell’s own, methylated DNA is protected from cleavage, while invading, unmethylated or differently methylated DNA is subjected to cleavage and subsequent removal from the cell.

*C*. *bescii* and *C*. *hydrothermalis* are so far the only species of *Caldicellulosiruptor* with an established genetic engineering toolbox. The development of this toolbox started with the identification of a potent HaeIII-like R-M system in *C*. *bescii*, which consists of the restriction enzyme CbeI and the corresponding methyltransferase M.CbeI [30, 31]. It was shown that methylation of plasmid DNA (isolated from a *dam*^*+*^ *dcm*^*+*^ *Escherichia coli* strain) with M.CbeI is required for transformation of *C*. *bescii*, although transformation efficiencies were extremely low [30]. It was also shown that deletion of *cbeI* in *C*. *bescii* allowed transformation of the strain without the requirement for prior *in vitro* methylation of the plasmid DNA by M.CbeI [32]. The tools for transformation of *C*. *bescii* have been successfully adapted to *C*. *hydrothermalis*, which was possible because both species contain similar R-M systems [33, 34].

In a previous study, we isolated several *Caldicellulosiruptor* strains from nature and characterized them with regard to their ability to ferment various substrates [35]. A few of the isolated strains produced lactic acid as the main fermentation product, and this industrially relevant characteristic was further improved in the last years by means of evolutionary adaptation [36]. Our current best available *Caldicellulosiruptor* strain produces 70 g/L of L-lactic acid from microcrystalline cellulose. Although evolutionary adaptation allowed us to significantly improve the L-lactic acid production from 6 to 70 g/L, the availability of a genetic engineering toolbox for the strain would accommodate us with a parallel path for further improvement using rational approaches. For reference, typical lactic acid concentrations from pretreated and hydrolysed lignocellulosic substrates reported in literature are 7–93 g/L for various lactic acid bacteria [37, 38], 40–134 g/L for *Bacillus coagulans* [39, 40], and 34–60 g/L for *Rhizopus oryzae* [41]. Lactic acid concentrations from easily accessible glucose or starch are generally much higher, reaching up to 210 g/L [39, 41].

In this study, we describe the identification of a novel SfaNI-like R-M system in the genus *Caldicellulosiruptor*. The identified system consists of a restriction endonuclease and two methyltransferases that recognize the reverse-complement sequences 5’-GATGC-3’ and 5’-GCATC-3’. We show that methylation of a DNA fragment by the methyltransferases is sufficient for transformation of *Caldicellulosiruptor* sp. strain DIB 104C. This strain and especially its descendants obtained by evolutionary adaptation [35, 36] produce high concentrations of L-lactic acid from microcrystalline cellulose and are therefore promising candidates for industrial valorisation.

## Materials and methods

### Strains, media and cultivation conditions

All strains used and constructed in this study are listed in Table 1.

**Table 1:** List of strains used and constructed in this study.

Strain	Reference
*Caldicellulosiruptor bescii* DSM 6725	DSMZ
*Caldicellulosiruptor acetigenus* DSM 7040	DSMZ
*Caldicellulosiruptor saccharolyticus* DSM 8903	DSMZ
*Caldicellulosiruptor lactoaceticus* DSM 9545	DSMZ
*Caldicellulosiruptor kristjanssonii* DSM 12137	DSMZ
*Caldicellulosiruptor owensensis* DSM 13100	DSMZ
*Caldicellulosiruptor hydrothermalis* DSM 18901	DSMZ
*Caldicellulosiruptor kronotskyensis* DSM 18902	DSMZ
*Caldicellulosiruptor changbaiensis* DSM 26941	DSMZ
*Caldicellulosiruptor danielli* DSM 8977	DSMZ
*Caldicellulosiruptor morganii* DSM 8990	DSMZ
*Caldicellulosiruptor naganoensis* DSM 8991	DSMZ
*Caldicellulosiruptor* sp. DIB 104C	[35]
*Caldicellulosiruptor* sp. DIB 104C D*pyrE*	This study
*Caldicellulosiruptor* sp. DIB 104C D*pyrE*::P_slp_-*pyrE*	This study
*Caldicellulosiruptor* sp. DIB 104C D*pyrE*::*pyrE*	This study
*Caldicellulosiruptor* sp. DIB 104C BluCon085	[36]

The MOPS-buffered medium with glucose used for routine cultivation of *Caldicellulosiruptor* cells contained per liter: D-glucose, 5 g; NH_4_Cl, 1 g; NaCl, 0.5 g; MgSO_4_ x 7 H_2_O, 0.3 g; CaCl_2_ x 2 H_2_O, 0.05 g; NaHCO_3_, 0.5 g; KH_2_PO_4_, 0.1 to 1 g; K_2_HPO_4_, 0.1 to 1 g; yeast extract, 2 g; MOPS, 1 to 20 g; resazurin, 0.25.10^−3^ g; vitamin solution, 5 mL; and trace element solution, 1 mL. The vitamin solution contained per liter: biotin, 4 mg; folic acid, 4 mg; pyridoxine hydrochloride, 20 mg; riboflavin, 10 mg; thiamine, 10 mg; nicotinic acid, 10 mg; pantothenic acid, 10 mg; vitamin B12, 0.2 mg; p-aminobenzoic acid, 10 mg; and thioctic acid, 10 mg. The trace element solution contained per liter: HCl (25%; 7.7 M), 10 mL; NiCl_2_ x 6 H_2_O, 0.024 g; FeCl_2_ x 4 H_2_O, 1.5 g; MnCl_2_ x 4 H_2_O, 0.1 g; CoCl_2_ x 6 H_2_O, 0.19 g; ZnCl_2_, 0.07 g; CuCl_2_ x 2 H_2_O, 0.002 g; H_3_BO_3_, 0.006 g; and Na_2_MoO_4_·2 H_2_O, 0.036 g. After dissolving all compounds in water, the pH of the medium was adjusted to 7.2 with 5 M NaOH. The medium was then flushed with N_2_ for 20 minutes, after which L-cysteine was added to a final concentration of 0.5 g/L. The medium was subsequently aliquoted in Hungate tubes [42] or serum bottles that had been flushed with N_2_ before. The medium was sterilized by autoclaving at 121°C and 1 bar overpressure for 20 minutes. Cultures of *Caldicellulosiruptor* cells were prepared by inoculation of the sterile medium with 0.25 to 1 mL of seed culture. The inoculation was performed under sterile and anaerobic conditions by injection with a syringe through the septum of the Hungate tube or serum bottle. The cultures were subsequently incubated in an orbital shaker at 100 rpm and 70°C.

The LOD medium used for the generation of electrocompetent *Caldicellulosiruptor* cells and the recovery of cells after transformation was made according to Farkas *et al*. [43]. For solid medium, 30 g/L of agar were added.

The MOPS-buffered medium with filter paper and CSM-Ura used for the selection of uracil prototrophic cells contained per liter: D-glucose, 0.5 g; (NH_4_)_2_SO_4_, 1.2 g; MgSO_4_ x 7 H_2_O, 0.3 g; CaCl_2_ x 2 H_2_O, 0.05 g; NaHCO_3_, 0.5 g; K_2_HPO_4_, 0.1 to 1 g; KH_2_PO_4_, 0.1 to 1 g; CSM-Ura, 0.77 g; MOPS, 1 to 20 g; resazurin, 0.25.10^−3^ g; vitamin solution, 5 mL; and trace element solution, 1 mL. After dissolving all compounds in water, the pH of the medium was adjusted to 7.2 with 5 M NaOH. Before transferring the medium into Hungate tubes, a strip of 1 to 6 cm of Whatman#1 filter paper (corresponding to approximately 50 mg) was placed in the tubes. The tubes were subsequently flushed with N_2_, closed with a septum, and incubated at room temperature for approximately one hour to remove the oxygen from the filter paper. Subsequent flushing of the medium with N_2_ and sterilization by autoclaving was performed in the same way as described for the MOPS-buffered medium with glucose.

The MOPS-buffered medium with glucose and CSM-Ura contained the same compounds as the MOPS-buffered medium with filter paper and CSM-Ura, with the only difference that 5 g/L of D-glucose was added instead of the filter paper.

The LB medium used for routine cultivation of *Escherichia coli* cells contained per liter: tryptone, 10 g; yeast extract, 5 g; and NaCl, 10 g. For solid medium, 15 g of agar were added. After dissolving all compounds in water, the medium was sterilized by autoclaving at 121°C and 1 bar overpressure for 20 minutes. Cultures of *E*. *coli* cells were prepared by inoculation of the sterile medium with cells from a single cell colony picked from plate. The cultures were subsequently incubated in an orbital shaker at 200 rpm and 37°C.

### General molecular biology methods

Genomic DNA of *Caldicellulosiruptor* cells was isolated using the MasterPure Gram Positive DNA Purification Kit from Epicentre Biotechnologies (Wisconsin, United States) according to the manufacturer’s instructions. Plasmid DNA of *E*. *coli* cells was isolated using the QIAprep Spin Miniprep Kit from Qiagen (Hilden, Germany).

Chemically competent *E*. *coli* cells were purchased from Invitrogen (Massachusetts, United States). Transformation of plasmid DNA into these cells was performed using the heat shock method described by Sambrook and Russell [44].

All restriction endonucleases were purchased from New England BioLabs (Massachusetts, United States), and restriction digests were performed according to the manufacturer’s instructions. Restriction products were separated on a 1% (w/v) agarose gel by electrophoresis and visualized by staining with SYBR Safe DNA Gel Stain (Invitrogen). Sizing of the restriction products was done using the GeneRuler 1 kb DNA Ladder from Thermo Fisher Scientific (Massachusetts, United States).

Polymerase chain reaction (PCR) was performed with Taq DNA Polymerase for diagnostic purposes, and Q5 High-Fidelity DNA Polymerase for genetic manipulation and sequencing purposes (New England BioLabs). All primers were ordered at biomers.net (Ulm, Germany). Purification of amplified DNA from the reaction mixtures was carried out using the QIAquick PCR Purification Kit (Qiagen).

Sequencing was carried out using the Sanger method at Eurofins (Luxembourg, Luxembourg). Sequencing data were analysed with Clone Manager version 9 (Sci Ed Software, Colorado, United States).

### Whole-genome sequencing

Whole-genome nucleotide sequences of strains DIB 104C and BluCon085 were obtained by combining Illumina and Nanopore sequencing reads to create a hybrid genome assembly. Illumina sequencing was done by LGC Genomics (Berlin, Germany) using an Illumina NextSeq500/550 V2 Sequencer, which generated 10 million read pairs (150 bp paired end) for each sample. Nanopore sequencing was done by first constructing long-read sequencing libraries with an LSK109 sequencing kit and subsequently loading them into an R9 flow cell for the MinION FLO-MIN106 platform (Oxford Nanopore Technologies, Oxford, United Kingdom). Sequence data collection during 6 hours generated 388 Mbp distributed on 24.5k reads for strain DIB 104C and 194 Mbp distributed on 84.8k reads for strain BluCon085. To generate the final whole-genome nucleotide sequences, the Nanopore sequencing reads were first assembled using the Flye software [45] and the draft sequences were then polished using the Illumina reads in combination with the Pilon software [46]. The final assembled and polished whole-genome nucleotide sequences of the strains DIB 104C and BluCon085 consisted of single contigs of about 2.6 Mbp. Genome annotation was conducted with Prokka software [47].

### Identification of the genomic methylation pattern using Tombo software package

Analysis, visualization and identification of modified nucleotides from Nanopore sequencing data was carried out with Tombo software package version 1.5 (Oxford Nanopore Technologies) [48]. This was done by motif-centered statistic plotting where genomic regions centered on a motif of biological interest are visualized by violin plots.

### Construction of plasmids

The construction of plasmids pTrc-Ec_cal02329M1 and pTrc-Ec_cal02329M2 started with the digestion of plasmid pTrcHis2 B (Thermo Fisher Scientific) with the restriction endonucleases NcoI and PmeI, which generated a fragment of 4,277 bp and a fragment of 127 bp. The larger fragment was subsequently purified from the reaction mixture using the QIAquick Gel Extraction Kit (Qiagen). In parallel to this work, two customized DNA constructs were ordered at GeneArt (Thermo Fisher Scientific) that consisted of the open reading frames of the genes *cal02329M1* and *cal02329M2*, codon-optimized for expression in *E*. *coli*. The open reading frames were flanked with 20-bp sequences complementary to the regions flanking the NcoI and PmeI recognition sequences in the plasmid pTrcHis2 B. The two customized DNA constructs were cloned into the purified 4,277-bp fragment of plasmid pTrcHis2 B using the GeneArt Gibson Assembly Cloning Kit (Thermo Fisher Scientific). Next, the reaction mixtures were used to transform chemically competent cells from *E*. *coli* strain NEB 10-beta, and transformants were selected on LB medium containing 100 μg/mL of ampicillin. Plasmids were subsequently extracted from *E*. *coli* cells using the QIAprep Spin Miniprep Kit. The integration of the genes *cal02329M1* and *cal02329M2* into the plasmid pTrcHis2 B was checked by restriction digestion, and the sequence of the genes was verified by sequencing. The maps of the plasmids pTrc-Ec_cal02329M1 and pTrc-Ec_cal02329M2 are shown in S1 Fig and the sequences of the codon-optimized genes *cal02329M1* and *cal02329M2* are shown in S2 Fig.

The construction of plasmid pUC19-P_slp_-pyrE started with the digestion of plasmid pUC19 (Thermo Fisher Scientific) with the restriction endonucleases BamHI and XbaI. The larger fragment of 2,680 bp was subsequently purified from the reaction mixture using the QIAquick Gel Extraction Kit. In parallel to this work, the fragments 5’ CIS1 (primers BLU03/04), 3’ CIS1 (primers BLU05/06), P_slp_ (primers BLU07/08) and *pyrE* (BLU09/10) were PCR amplified from genomic DNA of strain DIB 104C using Q5 High-Fidelity DNA Polymerase. All primers except BLU08 and BLU09 contain at their 5’ terminal end a recognition sequence for a specific restriction endonuclease (S1 Table). Primer BLU08 contains at its 5’ terminal end a sequence that is complementary to the 5’ terminal end of the *pyrE* open reading frame (S1 Table). After PCR amplification, all fragments were purified from the reaction mixtures using the QIAquick PCR Purification Kit. The fragments P_slp_ and *pyrE* were then linked to each other by means of Overlap Extension PCR [49], after which the fragment P_slp_-*pyrE* was purified from the reaction mixture using the QIAquick Gel Extraction Kit. All fragments were subsequently digested with the appropriate restriction endonucleases: 5’ CIS1 with BamHI and EcoRI, 3’ CIS1 with SalI and XbaI, and P_slp_-*pyrE* with EcoRI and SalI. The fragments were cloned into the purified 2,680-bp fragment of plasmid pUC19 using T4 DNA ligase (Thermo Fisher Scientific). Next, the reaction mixture was used to transform chemically competent cells from *E*. *coli* strain DH5a, and transformants were selected on LB medium containing 100 mg/mL of ampicillin. Plasmids were subsequently extracted from *E*. *coli* cells and checked by restriction digestion and sequencing. The map of the plasmid pUC19-P_slp_-pyrE is shown in S1 Fig and the sequence of the construct consisting of 5’ CIS1, P_slp_-*pyrE* and 3’ CIS1 is shown in S2 Fig.

### Production of methyltransferases M1.Cal02329 and M2.Cal02329 by heterologous expression in *E*. *coli* strain NEB 10-beta

Cells from a single cell colony of *E*. *coli* NEB 10-beta strains harbouring expression vectors pTrc-cal02329M1 and pTrc-cal02329M2, respectively, were used to inoculate 5 mL of LB medium containing 100 μg/mL of ampicillin. The cells were subsequently incubated overnight in an orbital shaker at 250 rpm and 37°C. An appropriate amount of cell culture to obtain an optical density at 600 nm (OD_600nm_) of 0.1 was transferred into 50 mL of LB medium containing 100 mg/ml of ampicillin, and incubated in an orbital shaker at 250 rpm and 37°C until an OD_600nm_ of 0.4 was reached. Then, expression of the methyltransferases M1.Cal02329 and M2.Cal02329 was induced by addition of 0.5 mM IPTG and incubation in an orbital shaker at 250 rpm and 28°C. The next day, an appropriate amount of cell culture to obtain an OD_600nm_ of 50 in 1 mL was transferred into a 50-mL tube, and cells were collected by centrifugation for 10 minutes at 4,000 x g and 10°C. The cell pellets were then stored at -20°C.

One cell pellet for each methyltransferase was resuspended in 1 mL of ice-cold lysis buffer. The lysis buffer contained per 40 mL: 1 tablet of cOmplete EDTA-free protease inhibitor cocktail (Roche, Basel, Switzerland), 4 mL of 0.5 M EDTA, 0.04 mL of 1 M DTT, and 4 mL of 10x CutSmart buffer (New England BioLabs). The cells were subsequently lysed using a Branson Sonifier 250. After the lysis process, the cell debris were removed by centrifugation for 5 minutes at 14,000 rpm and room temperature. The supernatants were transferred into 1.5-mL reaction tubes, and subsequently incubated for 10 minutes at 70°C to denature *E*. *coli* proteins. The denatured proteins were then removed by centrifugation for 5 minutes at 14,000 rpm. The supernatants containing the purified methyltransferases were transferred into new 1.5-mL reaction tubes, and used immediately for methylation of DNA.

### Validation of the heterologous expression of methyltransferases M1.Cal02329 and M2.Cal02329 by SDS-PAGE

Samples for SDS-PAGE were prepared by mixing 20 μL of cell lysate (either or not purified by heat treatment) with 8 μL of NuPAGE LDS Sample Buffer (4x) (Thermo Fisher Scientific), 3.2 μL of 0.5 M DTT and 0.8 μL of water. The samples were then boiled at 98°C for 10 min. In total 16 μL of each sample were loaded on a NuPAGE 4–12% Bis-Tris Gel (Thermo Fisher Scientific). As a reference, 5 μL of peqGOLD Protein Marker IV protein ladder (VWR International, Darmstadt Germany) was also loaded on the gel. Electrophoresis was performed in NuPAGE MES SDS Running Buffer (Thermo Fisher Scientific) using a vertical Invitrogen XCell SureLock Mini-Cell system (Thermo Fisher Scientific) at a voltage of 150. Staining of the proteins was performed using a 2% (v/v) Coomassie Brilliant Blue R-250 solution prepared in fixing solution (10% (v/v) glacial acid, 40% (v/v) ethanol, 50% (v/v) water), applying gentile agitation at room temperature. Destaining was done in fixing solution and followed over time by visual inspection.

### *In vitro* methylation of DNA using the methyltransferases M1.Cal02329 and M2.Cal02329

*In vitro* methylation of DNA was performed by mixing the following compounds in a reaction tube: 10 μg of DNA in a volume of 20 μL, 10 μL of purified M1.Cal02329 methyltransferase, 10 μL of purified M2.Cal02329 methyltransferase, and 1 μL of S-adenosyl-methionine (32 mM). The mixture was then incubated at 70°C for 6 hours. After each hour of incubation, 1 μL of S-adenosyl-methionine was added to the mixture, and after 3 hours of incubation, 10 μL of each methyltransferase was added.

To purify the methylated DNA from the mixture, the volume of the mixture was first adjusted to 300 μL using water in a 2-mL reaction tube. Then, 300 μL of phenol:chloroform:isoamylalcohol (PCI; 25:24:1) was added and mixed with the methylation reaction mixture by thoroughly inverting the tube for 1 minute. After 10 minutes of centrifugation at 14,000 rpm and room temperature, 250 μL of the upper layer containing the DNA were transferred into a new 2-mL reaction tube, and 250 μL of PCI was added. After mixing for 1 minute, the mixture was once again centrifuged at 14,000 rpm and room temperature. In total 200 μL of the upper layer were transferred into a 1.5-mL reaction tube. Next, 200 μL of isopropanol and 20 μL of sodium acetate (3 M) were added to the mixture, and the solution was mixed by inverting the reaction tube vigorously. After 30 minutes of incubation at room temperature, the precipitated DNA was collected by centrifugation for 15 minutes at 14,000 rpm and room temperature. The DNA pellet was then washed with 500 μL of 70% (v/v) ethanol, and subsequently dried at room temperature. The DNA pellet was finally resuspended in 50 μL of water.

### Preparation of a cell lysate from strain BluCon085

Cells from strain BluCon085 were used to inoculate 500 mL of MOPS-buffered medium with glucose to an OD_600nm_ of 0.1. The culture was subsequently incubated in an orbital shaker at 100 rpm and 70°C until mid-logarithmic phase (OD_600nm_ of approximately 0.8) was reached. The cells were then harvested by centrifugation for 15 minutes at 6,000 x g and 4°C, and resuspended in 500 μl of CelLytic B Cell Lysis Reagent (Sigma-Aldrich, Missouri, United States) containing cOmplete EDTA-free protease inhibitor cocktail. The cell suspension was subsequently sonicated on ice using a Branson Sonifier 250. The cell lysate was then centrifuged for 15 minutes at 14,000 rpm and 4°C to remove the cell debris.

The supernatant was used immediately to assess degradation of a DNA fragment by endogenous nucleases expressed by strain BluCon085. The reactions were performed in a final volume of 14 μl containing 0.5 μg of DNA and either 14% (2 μl), 29% (4 μl) or 43% (6 μl) of cell lysate. Adjustment of the volume to the final volume was performed with CutSmart buffer containing 1 mM of DTT. The reaction mixtures were subsequently incubated at 70°C for 30 minutes. After incubation, the restriction products were separated on a 1% (w/v) agarose gel by electrophoresis and visualized by staining.

### Transformation of strain DIB 104C D*pyrE* by electroporation

With exception of the centrifugation steps and the pulse delivery for electroporation, all working steps for the preparation of electrocompetent cells and for the transformation were performed in an anaerobic workstation.

Electrocompetent cells of strain DIB 104C Δ*pyrE* were prepared according to Chung *et al*. [30]. In total 50 μL of the electrocompetent cell suspension were then transferred into a 1.5-mL reaction tube and mixed with 1 μg of DNA provided in 10 μL of water. After 15 minutes of incubation at room temperature, the cell suspension was transferred into an ice-cold electroporation cuvette (1 mm) for electroporation using a Gene Pulser from Bio-Rad Laboratories (California, United States). The electroporation parameters were as follows: single exponential pulse, field strength of 1 to 2 kV (10 to 20 kV/cm), resistance of 500 to 700 Ohm, and capacity of 20 to 50 μF. Immediately after electroporation, 500 μL of LOD medium were transferred into the cuvette and the cell suspension was transferred into a Hungate tube containing 9 mL of LOD medium with 40 μM of uracil. The culture was incubated overnight in an orbital shaker at 70°C and 100 rpm.

After the overnight regeneration, the cells were collected by centrifugation for 15 minutes at 4,000 x g and room temperature, and the cell pellet was washed in 2 mL of MOPS-buffered medium with CSM-Ura. After another centrifugation step, the cell pellet was resuspended in 1 mL of MOPS-buffered medium with CSM-Ura. Then, 100 μL of the cell suspension were transferred into a Hungate tube containing 9 mL of MOPS-buffered medium with filter paper and CSM-Ura for selection of prototrophic cells; another 100 μL were transferred into the same medium supplemented with 40 μM of uracil for control of cell viability. The cultures were subsequently incubated in an orbital shaker at 100 rpm and 70°C.

After decomposition of the filter paper was observed, an aliquot of the culture was streaked on a plate containing solid LOD medium to obtain single cell colonies. The plate was placed in an anaerobic jar with a Millipore Anaerocult A tablet (Merck, Darmstadt, Germany), and incubated in a static incubator at 70°C until single cell colonies were observed.

## Results

### Strain BluCon085 shows a different restriction endonuclease pattern as compared to *C*. *bescii* strain DSM 6725

Chung *et al*. [32] have digested genomic DNA of type strains of seven *Caldicellulosiruptor* species with several restriction endonucleases, and showed a difference in susceptibility to HaeIII. The relevance of HaeIII relates to the fact that *C*. *bescii* strain DSM 6725 has a potent restriction endonuclease that is an isoschizomer of HaeIII cleaving unmethylated sequences at 5´-GGCC-3´ [31]. The methyltransferase related to the restriction endonuclease methylates DNA at the m4C position within this sequence [30].

In the current study, HaeIII was applied in a reaction with genomic DNA of strain BluCon085. Strain BluCon085 is a derivative from strain DIB 104C that was obtained by evolutionary adaptation of the latter strain for production of high concentrations of L-lactic acid [35, 36]. Our data show that genomic DNA of strain BluCon085 is digested by HaeIII (Table 2), implying that the sequence 5’-GGCC-3’ is not methylated in this strain and thus a different R-M system must be active as compared to *C*. *bescii* strain DSM 6725.

**Table 2:** Digestion of genomic DNA of type strains of *Caldicellulosiruptor* species with the restriction endonucleases HaeIII and SfaNI.

Strain	Restriction endonuclease	
	HaeIII	SfaNI
*C*. sp. DIB 104C	+	-
*C*. sp. DIB 104C BluCon085	+	-
*C*. *bescii* DSM 6725	-	+
*C*. *acetigenus* DSM 7040	+	+
*C*. *saccharolyticus* DSM 8903	-	-
*C*. *lactoaceticus* DSM 9545	+	+
*C*. *kristjanssonii* DSM 12137	-	+
*C*. *owensensis* DSM 13100	+	+
*C*. *hydrothermalis* DSM 18901	-	+
*C*. *kronotskyensis* DSM 18902	+	+
*C*. *changbaiensis* DSM 26941	+	-
*C*. *danielli* DSM 8977	+	+
*C*. *morganii* DSM 8990	+	+
*C*. *naganoensis* DSM 8991	+	-

The “+” sign indicates that the genomic DNA is digested by the restriction endonuclease; the “-”sign indicates that the genomic DNA is not digested. Pictures of the corresponding agarose gels are shown in the supporting information (S3 Fig).

### Identification of a putative R-M system in strain BluCon085

The phylogenetic tree of *Caldicellulosiruptor* strains published by Svetlitchnyi *et al*. [35] shows that strain DIB 104C (and by extension also strain BluCon085) is most closely related to the type strain *C*. *saccharolyticus* DSM 8903. For the latter strain, two putative R-M systems are described in REBASE, which are assigned to the classification Type I and Type IIS [50]. The putative Type I R-M system consists of the restriction endonuclease CsaDORF2680P, the methyltransferase M.CsaDORF2680P, and the specificity subunit S.CsaDORF2680P. The recognition sequence and the methylation site of the respective enzymes have not yet been identified. The putative Type IIS R-M system consists of the restriction endonuclease CsaDORF2754P and the methyltransferases M1.CsaDORF2754P and M2.CsaDORF2754P. The recognition sequence of the system is 5’-GASTC-3’, whereas the methylation site within this sequence has not yet been identified.

The restriction endonuclease CsaDORF2754P is a member of the AlwI family Type IIS restriction endonucleases, which includes AlwI (5’-GGATC-3’), Bsp6I (5’-GCNGC-3’), BstNBI (5’-GASTC-3’), PleI (5’-GAGTC-3’) and MlyI (5’-GAGTC-3’) [51]. Restriction endonucleases from this family recognize asymmetric sequences and cleave outside of their recognition sequence [52]. The restriction endonucleases are related to two methyltransferases, of which one is specific for the top-strand sequence, and the other for the complementary bottom-strand sequence.

A tblastn analysis of the three enzymes from the putative Type IIS R-M system from *C*. *saccharolyticus* strain DSM 8903 against the whole-genome nucleotide sequence of strain BluCon085 identified proteins representing a homologous R-M system in this strain. The restriction endonuclease CsaDORF2754P (RefSeq accession number WP_011918235) shows 97.5% identity with the protein R.Cal02329, and the methyltransferases M1.CsaDORF2754P (WP_011918236) and M2.CsaDORF2754P (WP_011918237) show 97.8% and 98.8% identity with the proteins M1.Cal02329 and M2.Cal02329, respectively (S2 and S4 Figs). The genes encoding for R.Cal02329, M1.Cal02329 and M2.Cal02329 are clustered within an operon.

As strain DIB 104C and its derivative BluCon085 have not yet been assigned to a known species or classified as a new species, we could not adopt the guidelines from Roberts [53] for the naming of the identified restriction endonuclease and methyltransferases. Therefore, the naming has been done arbitrarily. The restriction endonuclease is referred to as R.Cal02329, and the methyltransferases as M1.Cal02329 and M2.Cal02329, in which the prefix “Cal” refers to the genus “*Cal**dicellulosiruptor”* and the suffix “02329” to the number of the ORF encoding for the restriction endonuclease. The genes encoding for the restriction endonuclease and methyltransferases are referred to as *cal02329R*, *cal02329M1*, and *cal02329M2*, respectively.

### Identification of the recognition sequences of the putative R-M system in strain BluCon085

**Fig 1:**
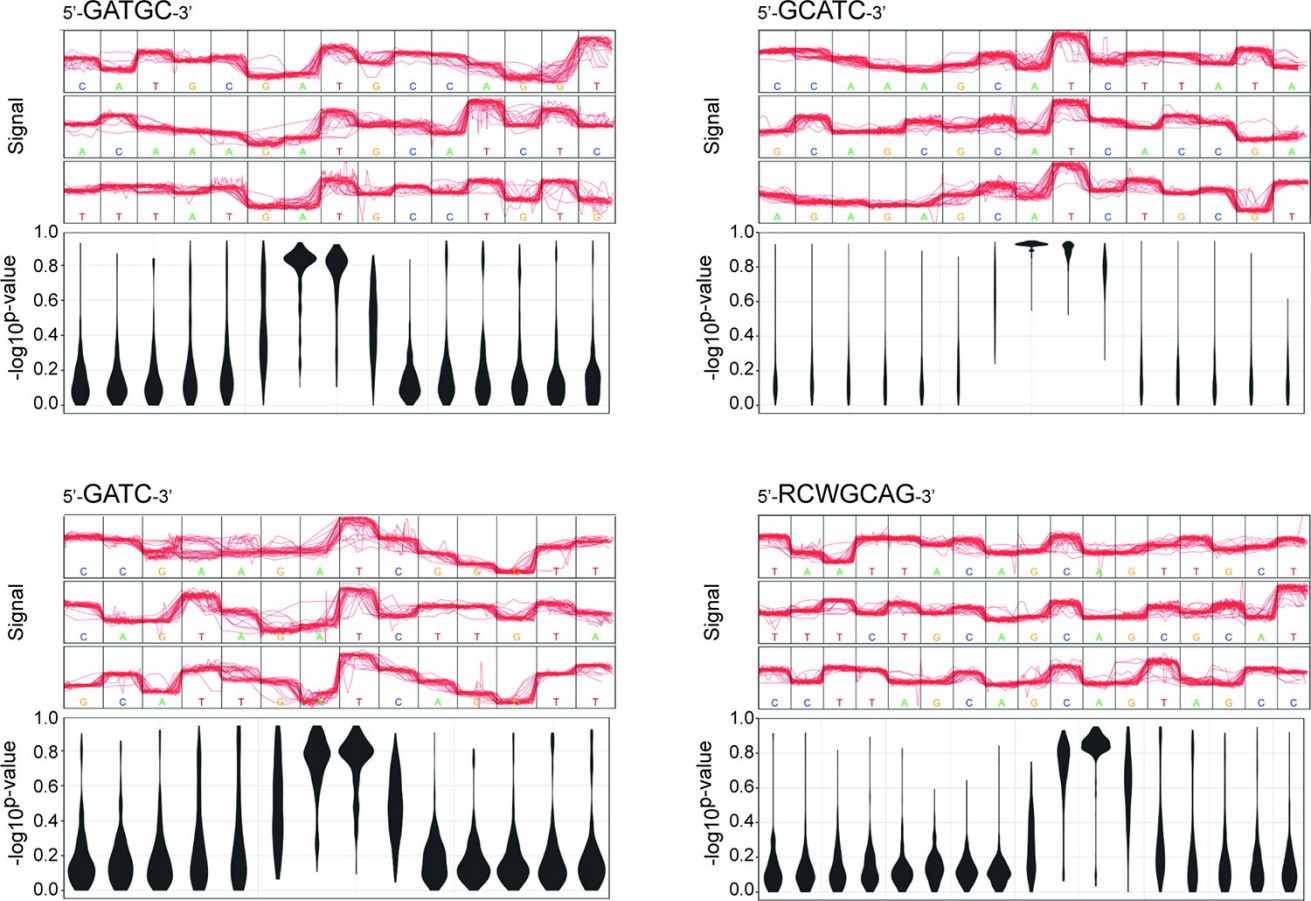
Prediction of the four most prominent DNA methylation patterns detected in the Nanopore sequencing dataset from strain BluCon085 using the methylation-calling tool Tombo version 1.5. For each identified methylation pattern, raw Nanopore signals (red lines) for three related sequence motifs in the genome and the corresponding violin plots are shown. The violin plots visualize the predicted methylation frequencies of single nucleotides (y-axis/-log10^p-value^) for related sequence motifs with a given proportion of methylated reads (x-axis/DNA sequence). Modified bases are highlighted in the violin plot by methylation frequencies greater than 0.8. The predicted methylated sites within the identified motifs are “A” and “T” in the related asymmetric sequences 5’-GATGC-3’ and 5’-GCATC-3’, “A” and “T” in the palindromic sequence 5’-GATC-3’, and “C” and “A” in the asymmetric sequence 5’-RCWGCAG-3’. As for the latter one, the methylated reverse-complement counterpart could not be detected, and therefore hemi-methylation is assumed.

The methylation pattern of the genomic DNA of strain BluCon085 was determined by analysing the Nanopore sequencing dataset using the Tombo software package [48]. The most prominent sequences in which modifications were identified are 5’-GATGC-3’ (3771 positions), 5’-GCATC-3’ (3771 positions), 5’-GATC-3’ (2095 positions), and 5’-RCWGCAG-3’ (1678 positions) (Fig 1). The predicted methylated sites within these sequences are “A” and “T” in 5’-GATGC-3’, “A” and “T” in 5’-GCATC-3’, “A” and “T” in 5’-GATC-3’, and “C” and “A” in 5’-RCWGCAG-3’. It is noted that the sequences 5’-GATGC-3’ and 5’-GCATC-3’ are reverse-complement sequences and have the same number of occurrences, implying that they constitute the same sites in the double-stranded genomic DNA.

**Fig 2:**
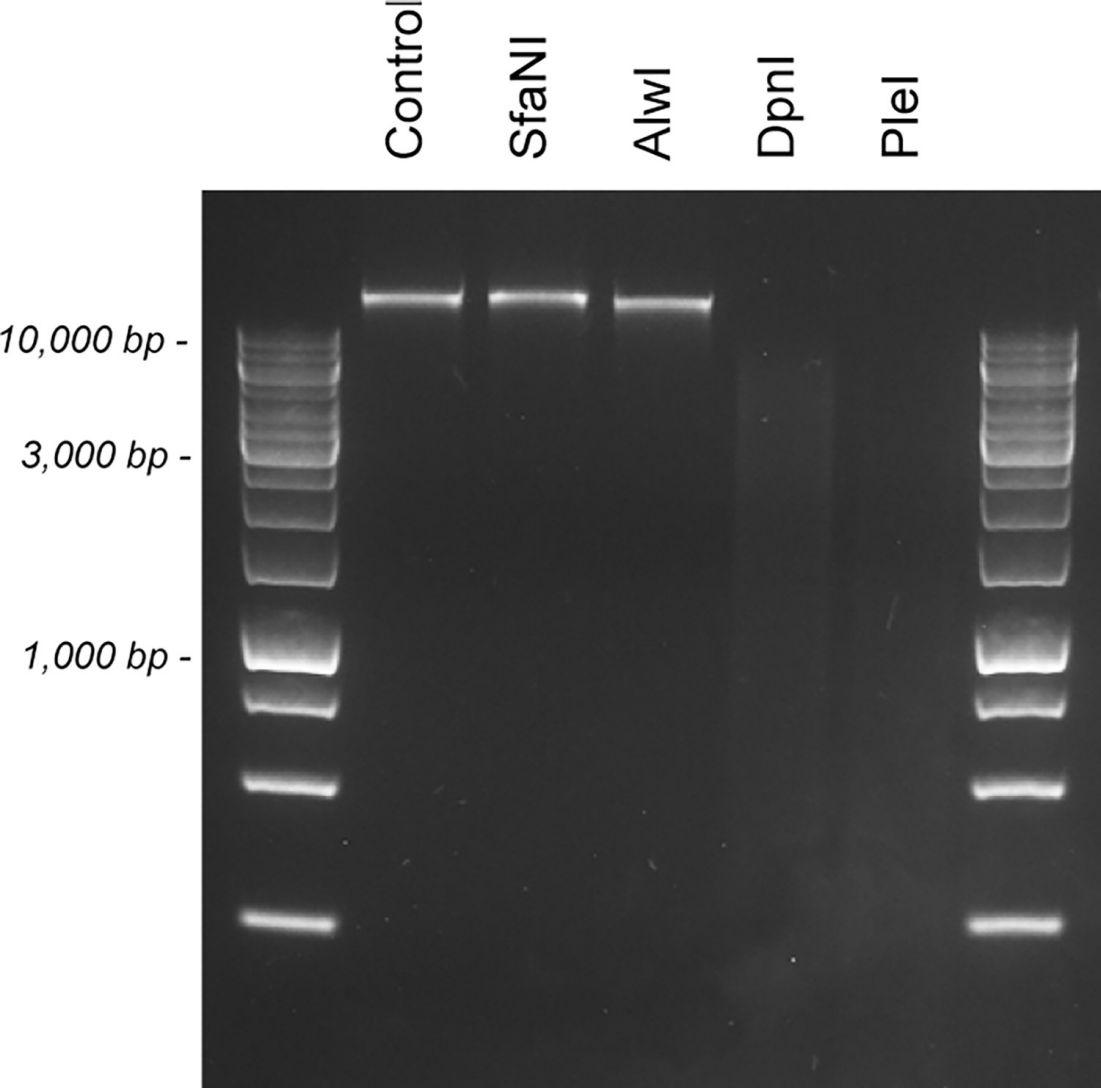
Digestion of genomic DNA of strain BluCon085 with methylation-sensitive restriction endonucleases. Restriction digests were performed by incubating genomic DNA in the presence of the restriction endonuclease SfaNI, AlwI, DpnI or PleI. A restriction digest without restriction endonuclease was included as control.

To experimentally verify the predicted methylated sites, genomic DNA of strain BluCon085 was digested with the restriction endonuclease SfaNI recognizing the sequence 5‘-GCATC-3‘, and the restriction endonucleases DpnI and AlwI recognizing the sequences 5’-GATC-3’ and 5‘-GGATC-3‘, respectively. DpnI is a methyl-directed restriction endonuclease that only cuts when its recognition sequence is methylated at the m6A position. Unfortunately, no enzyme that recognizes the sequence 5’-RCWGCAG-3’ is commercially available. Our data show that genomic DNA of strain BluCon085 is digested by the restriction endonuclease DpnI, but not by the restriction endonucleases AlwI and SfaNI (Fig 2). These data show that the three sequences 5’-GATGC-3’, 5’-GCATC-3’, and 5’-GATC-3’ identified by the Tombo software package are indeed methylated in the genomic DNA of strain BluCon085.

As the putative R-M system in strain BluCon085 is homologous to the Type IIS R-M system of *C*. *saccharolyticus* strain DSM 8903 with recognition sequence 5’-GASTC-3’, genomic DNA of strain BluCon085 was also digested with the restriction endonuclease PleI recognizing the sequence 5’-GAGTC-3’. Our data show that the genomic DNA of strain BluCon085 is digested by PleI (Fig 2), implying that the corresponding recognition sequence is not methylated.

To learn which of the above-mentioned methylated recognition sequences is linked to the restriction endonuclease R.Cal02329 in strain BluCon085, a blastp analysis was carried out in REBASE using the sequence of the protein. Among the top five restriction endonucleases most closely related to R.Cal02329 is PbaD1IIIP (30% identity) originating from *Phycisphaerae bacterium* SM-Chi-D1, which recognizes the sequence 5’-GCATC-3’. This sequence is identical to one of the sequences identified in the Nanopore sequencing dataset and confirmed by restriction endonuclease digestion of genomic DNA of strain BluCon085. The restriction endonuclease PbaD1IIIP belongs to the Type IIS R-M systems, and the methyltransferase related to PbaD1IIIP is known to modify the nucleobase adenine (m6A) [50].

### Methylation by M1.Cal02329 and M2.Cal02329 reduces degradation of DNA by endogenous nucleases present in a BluCon085 cell lysate

**Fig 3:**
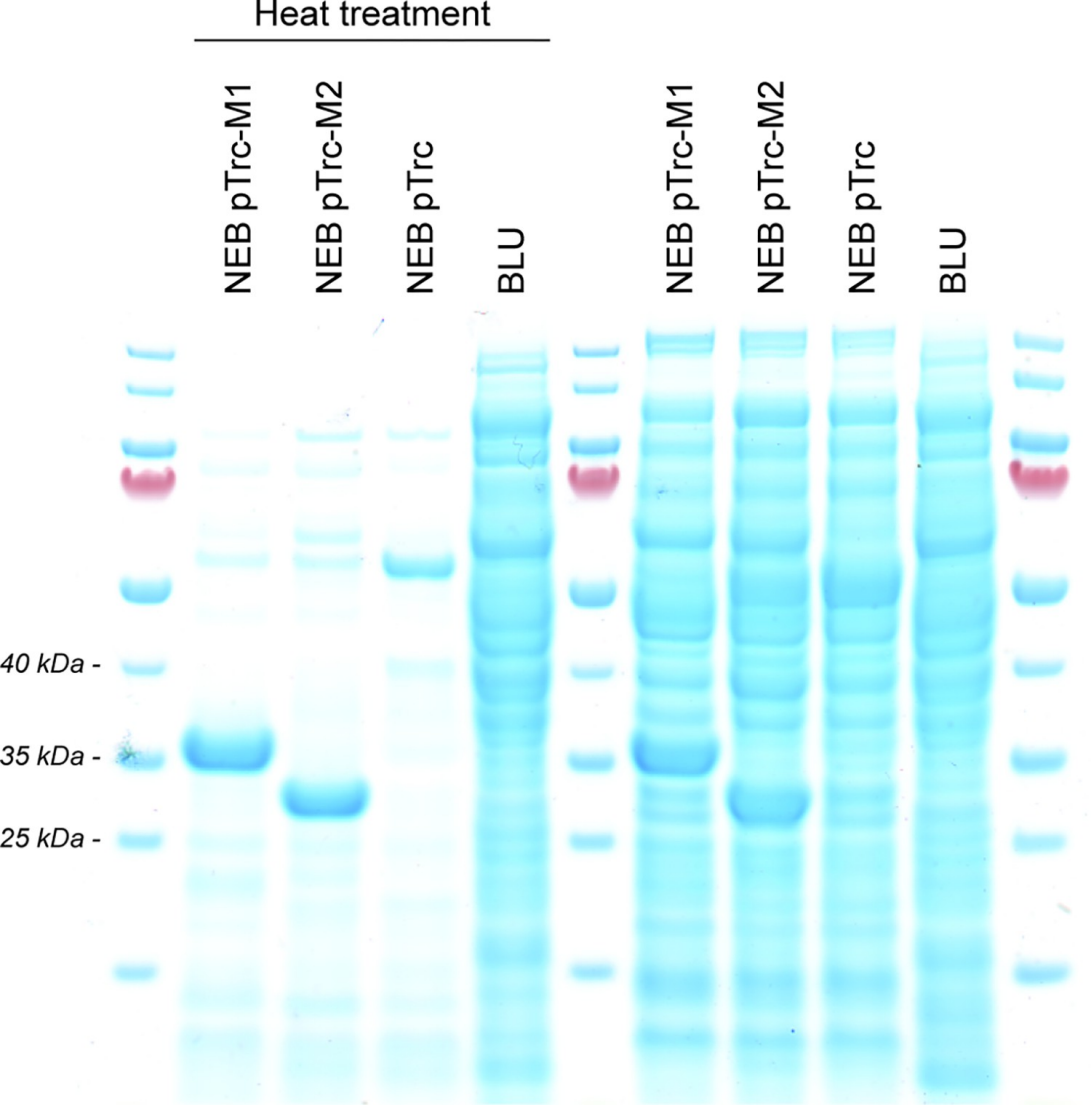
SDS-PAGE analysis of protein extracts from *E*. *coli* NEB 10-beta strains expressing methyltransferases M1.Cal02329 and M2.Cal02329. Protein extracts were prepared from *E*. *coli* strain NEB 10-beta pTrc-Ec_cal02329M1 (NEB pTrc-M1), *E*. *coli* strain NEB 10-beta pTrc-Ec_cal02329M2 (NEB pTrc-M2), *E*. *coli* strain NEB 10-beta pTrcHis2 B (NEB pTrc), and *Caldicellulosiruptor* strain BluCon085 (BLU). Cells were first lysed by sonification, and thermotolerant proteins present in the cell lysates were purified by heat treatment at 70°C. Protein extracts before and after purification are shown.

The open reading frames from the methyltransferases M1.Cal02329 and M2.Cal02329 were codon-optimized for expression in *E*. *coli* and cloned behind the Trc promoter into the expression vector pTrcHis2 B. The vectors were then transferred into *E*. *coli* strain NEB 10-beta, and expression of the methyltransferases was induced with IPTG. The cells were subsequently lysed, after which the cell lysates were incubated at 70°C to promote denaturation of *E*. *coli* proteins. An SDS-PAGE analysis of the cell lysates before and after purification of the methyltransferases shows the effectiveness of the purification step, as the bulk of the proteins are removed by the heat treatment (Fig 3). The analysis also shows the presence of two protein fractions with a molecular weight that corresponds to the predicted molecular weight of the methyltransferases M1.Cal02329 and M2.Cal02329 (31.5 and 28.5 kDa, respectively) (Fig 3). Neither one of the two protein fractions is present in the purified cell lysate from *E*. *coli* strain NEB 10-beta harbouring the empty plasmid pTrcHis2 B.

**Fig 4:**
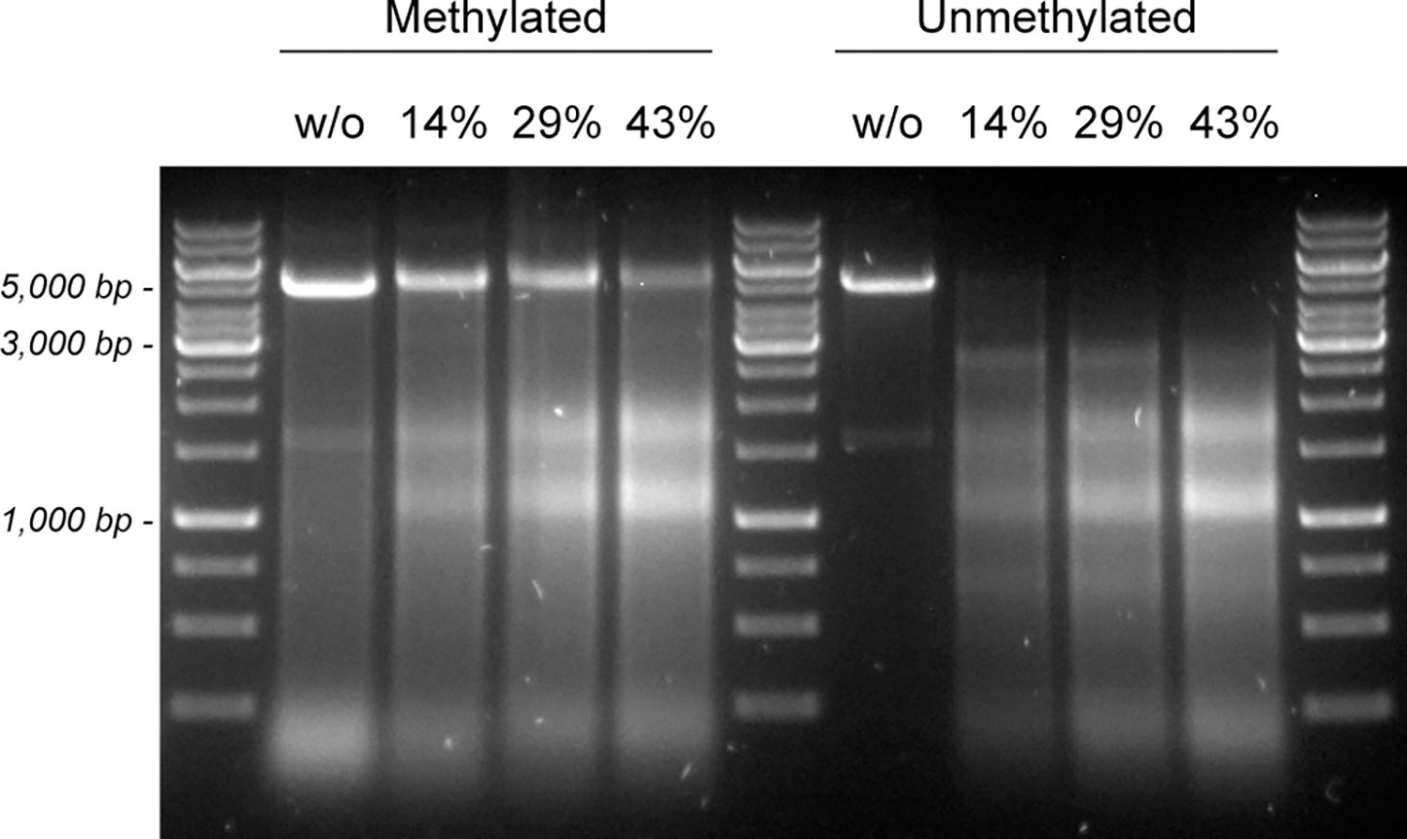
Degradation of a methylated and unmethylated DNA fragment by endogenous nucleases present in a BluCon085 cell lysate. The methylated and unmethylated DNA fragments were incubated in the absence of cell lysate (w/o), and in the presence of 14%, 29% and 43% of cell lysate. The incubation was performed at 70°C for 30 minutes.

The purified methyltransferases M1.Cal02329 and M2.Cal02329 were then used to methylate a DNA fragment that was obtained by PCR amplification from plasmid pUC19-P_slp_-pyrE. The primers used in the PCR were BLU01 and BLU02 (S1 Table), and the size of the amplified fragment was 4,946 bp. To check the effectiveness of the methylation reaction, the methylated DNA fragment was incubated with three different concentrations of cell lysate from strain BluCon085 to assess degradation of the fragment by endogenous nucleases. The reactions were performed at 70°C for 30 minutes. As a reference, the original, unmethylated DNA fragment was treated under the same conditions. Our data show that methylation of a DNA fragment using the methyltransferases M1.Cal02329 and M2.Cal02329 reduces degradation of the fragment by endogenous nucleases present in a BluCon085 cell lysate as compared to the unmethylated DNA fragment (Fig 4). The latter is completely degraded within the 30-minutes time frame of the experiment.

### Construction of a *pyrE* marker selection system for strain DIB 104C

A commonly used marker selection system in transformation experiments is based on the uridine monophosphate (UMP) metabolic pathway [54–58], in which UMP is produced from glutamate *via* six consecutive reactions. The last two reactions involve the conversion of orotic acid to orotidine 5’-monophosphate (OMP) by orotate phosphoribosyltransferase, and the conversion of OMP to UMP by orotidine 5’-phosphate decarboxylase. In *Caldicellulosiruptor*, orotate phosphoribosyltransferase and orotidine 5’-phosphate decarboxylase are encoded by the gene *pyrE* and *pyrF*, respectively. Besides the *de novo* synthesis from glutamate, UMP can also be synthesized from uracil taken up from the growth medium. In this case, uracil is directly converted to UMP by uracil phosphoribosyltransferase.

The marker selection system based on the UMP metabolic pathway relies on the generation of a mutant strain with a defect in the *de novo* UMP metabolic pathway, and the inability of this strain to grow on defined medium without uracil. In most cases, a mutant strain with a defect in the *pyrE* or *pyrF* gene is used.

**Fig 5:**
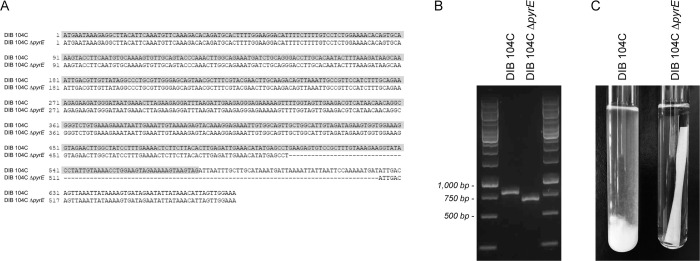
Confirmation of the deletion in the *pyrE* gene in strain DIB 104C D*pyrE*. (A) The *pyrE* open reading frame together with 100 bp immediately downstream of the stop codon was sequenced in strains DIB 104C and DIB 104C D*pyrE*. The *pyrE* open reading frame in strain DIB 104C is indicated in grey. (B) The *pyrE* gene was PCR amplified from genomic DNA of strains DIB 104C and DIB 104C D*pyrE* using primers BLU13/14 (S1 Table), which bind upstream and downstream of the *pyrE* open reading frame. (C) The uracil auxotrophy of strain DIB 104C D*pyrE* was checked by incubating cells in MOPS-buffered medium with filter paper and CSM-Ura. Incubation was performed at 70°C for 4 days.

In this study, a *pyrE* mutant version from strain DIB 104C was constructed by exposing cells to the compound 5-fluoroorotic acid (5-FOA), and selecting for spontaneous mutations that enable cells to grow in the presence of this compound. The selection pressure is based on the fact that 5-FOA is converted *via* two consecutive reactions catalysed by the enzymes PyrE and PyrF to 5-fluorouridine monophosphate, which is toxic to the cell and leads to cell death [54]. In particular, cells of strain DIB 104C were used to inoculate MOPS-buffered medium with glucose and 0.5 g/L of 5-FOA to an OD_600nm_ of 0.01. After 3 days of incubation at 70°C, an increase in the turbidity of the growth medium was observed. An aliquot of the culture was then streaked on solid MOPS-buffered medium with glucose and 0.1 g/L of 5-FOA and cells were grown at 70°C to obtain single cell colonies. In total 64 single cell colonies were isolated and directly checked for the occurrence of the *pyrE* and *pyrF* gene by means of PCR and sequencing. One strain showed a 114-bp deletion in the *pyrE* gene, of which 66 bp were deleted from the 3’ terminal end of the open reading frame (Fig 5A and 5B). The deletion generates a premature stop codon, resulting in a protein with 173 amino acids as compared to the wild type protein with 191 amino acids. The strain did not show any deletions or other mutations in the *pyrF* gene. Inoculation of MOPS-buffered medium with filter paper and CSM-Ura with cells from the *pyrE* mutant version from strain DIB 104C confirmed that they are not able to grow in the absence of uracil (Fig 5C).

### Methylation of DNA by M1.Cal02329 and M2.Cal02329 enables transformation of strain DIB 104C

To prove that methylation of a DNA fragment by the methyltransferases M1.Cal02329 and M2.Cal02329 enables transformation of strain DIB 104C, a transformation experiment was conceived in which a P_slp_-*pyrE* construct was introduced into the *pyrE* mutant version of the strain. P_slp_ is a promoter region that has been applied before to drive expression of various heterologous genes in *C*. *bescii* [9, 59, 60]. The expression of the *pyrE* gene under control of the P_slp_ promoter is expected to complement the uracil auxotrophy of strain DIB 104C D*pyrE*.

**Fig 6:**
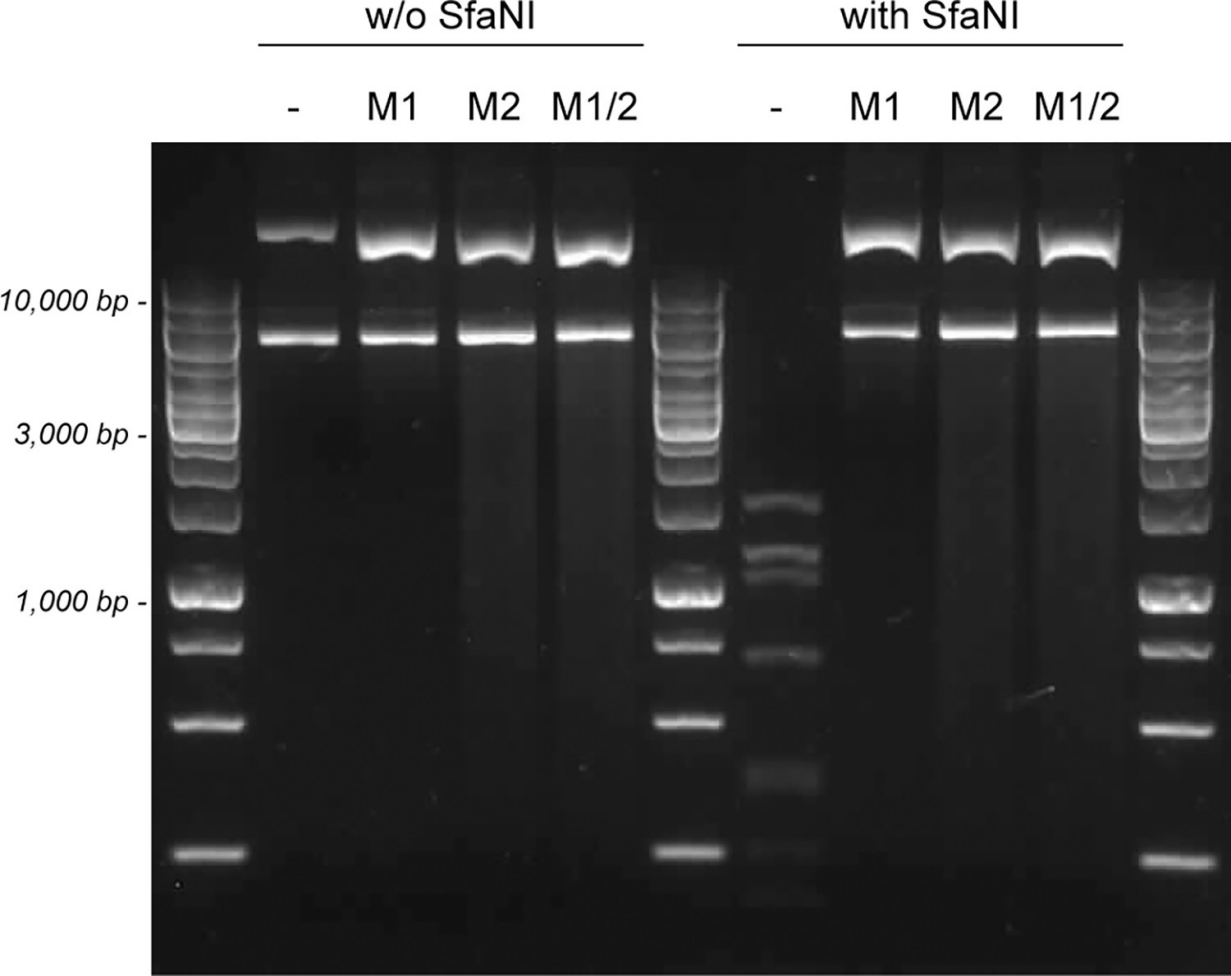
Digestion of methylated and unmethylated pUC19-P_slp_-pyrE plasmid DNA with the restriction endonuclease SfaNI. Restriction digests were performed by incubating unmethylated plasmid DNA (-) as well as plasmid DNA methylated with either M1.Cal02329 (M1), or M2.Cal02329 (M2), or both M1.Cal02329 and M2.Cal02329 (M1/2) in the presence of the restriction endonuclease SfaNI. A restriction digest without SfaNI was included as control.

First, a pUC19 plasmid was constructed in which a P_slp_-*pyrE* construct was placed in between two fragments of 991 bp and 1,011 bp that flank a probable neutral region in the genome of strain DIB 104C. The plasmid was then methylated in three parallel reactions: one reaction containing only M1.Cal02329, one containing only M2.Cal02329, and one containing both M1.Cal02329 and M2.Cal02329. The efficiencies of the methylation reactions were verified by digesting the methylated plasmids as well as the original, unmethylated plasmid with the restriction endonuclease SfaNI. Our data show that all methylated plasmids are protected from digestion with SfaNI, while the unmethylated plasmid is not protected (Fig 6). This implies that methylation with only one of the two methyltransferases is already sufficient for protection of a DNA fragment from digestion with SfaNI.

The pUC19-P_slp_-pyrE plasmid methylated with both methyltransferases was subsequently used to transform electrocompetent DIB 104C Δ*pyrE* cells by electroporation (three replicates). As control, the unmethylated plasmid was also included in the experiment. After an overnight recovery step, the cells were transferred into MOPS-buffered medium with filter paper and CSM-Ura. After 4 days of incubation at 70°C, decomposition of the filter paper was observed for cells transformed with the methylated plasmid DNA, while no decomposition was observed for cells transformed with the unmethylated DNA.

**Fig 7:**
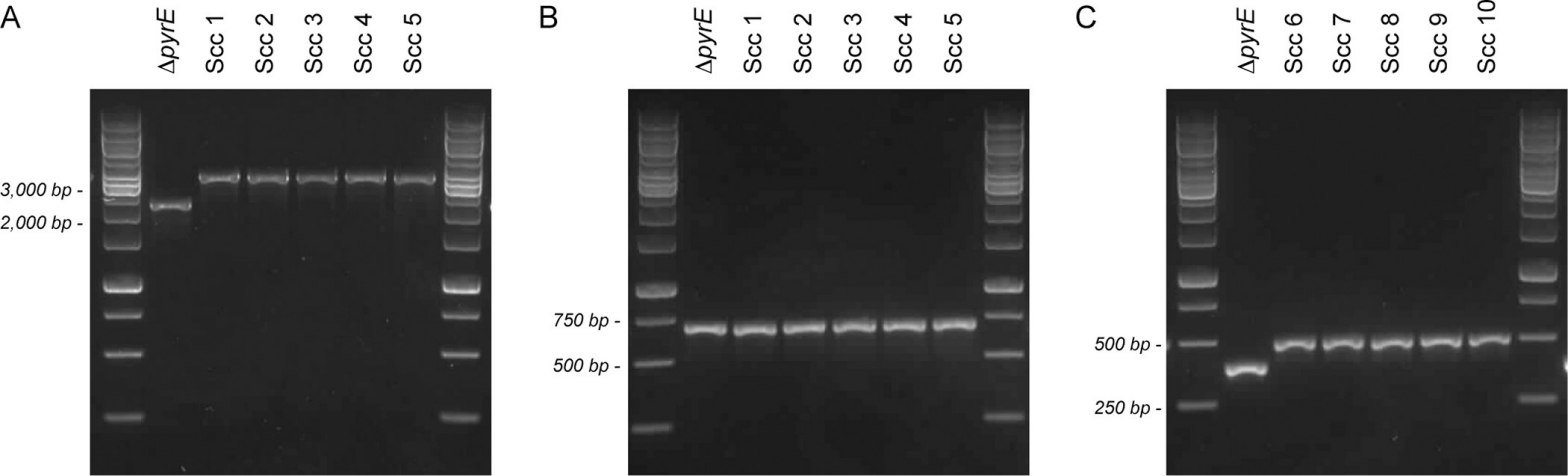
Confirmation of genomic integration of the *pyrE* gene by PCR. (A) Confirmation of the integration of a fragment in the CIS1 region in five single cell colonies (Scc 1 to Scc 5) obtained after transformation of strain DIB 104C D*pyrE* with plasmid pUC19-P_slp_-pyrE. Strain DIB 104C D*pyrE* was included as control (D*pyrE*). (B) Confirmation of the mutant version of the *pyrE* gene in the single cell colonies Scc 1 to Scc 5. (C) Confirmation of the wild type *pyrE* gene in five single cell colonies (Scc 6 to Scc 10) obtained after transformation of strain DIB 104C D*pyrE* with a linear DNA fragment containing the wild type *pyrE* gene.

From one culture showing decomposition of the filter paper, an aliquot was streaked on solid LOD medium to obtain single cell colonies. After 6 days of incubation at 70°C, cells from five single cell colonies were resuspended in MOPS-buffered medium with glucose and CSM-Ura. Next, genomic DNA was extracted from the cultures, and the genomic integration of the P_slp_-*pyrE* construct was checked by PCR using primer pair BLU11 plus BLU12 (S1 Table). Both primers bind to genomic DNA upstream and downstream of the 5’ CIS1 and 3’ CIS1 site, respectively. Our data show integration of a fragment in the CIS1 region in each of the five strains (Scc 1 to Scc 5) (Fig 7A). Sequencing of the entire CIS1 region shows the presence of the P_slp_-*pyrE* construct but not of the pUC19 backbone, implying that the P_slp_-*pyrE* construct was integrated *via* a double crossover event. The presence of the mutant version of the *pyrE* gene was confirmed by PCR using primer pair BLU13 plus BLU14, which bind genomic DNA upstream and downstream of the *pyrE* open reading frame (Fig 7B).

As integration of the P_slp_-*pyrE* construct at the CIS1 region occurred *via* a double crossover event, the question arose whether transformation could also be achieved by using a linear DNA fragment. Therefore, a second transformation experiment was conceived in which a linear DNA fragment comprising the wild type *pyrE* gene was introduced into strain DIB 104C Δ*pyrE*. The linear DNA fragment contained the *pyrE* open reading frame together with 1,005 bp immediately upstream and 925 bp immediately downstream of the start and stop codon, respectively. The fragment was obtained by PCR amplification on genomic DNA of strain DIB 104C using primer pair BLU15 plus BLU16 (S1 Table). Both primers contain three phosphorothioate modifications at their 5’ terminal end, aiding to protect the amplified product from exonuclease degradation.

The methylation, transformation, recovery and selection steps were performed in a similar manner as described above. After 5 days of incubation at 70°C, decomposition of the filter paper was observed for cells transformed with the methylated linear DNA. Genomic reversion of the mutant to the wild type *pyrE* gene was confirmed in five single cell colonies by PCR using primer pair BLU14 plus BLU17 (Scc 6 to Scc 10) (Fig 7C and S1 Table).

### Evidence for a SfaNI-like R-M system in other *Caldicellulosiruptor* species

The amino acid sequences of the restriction endonuclease R.Cal02329 and the methyltransferases M1.Cal02329 and M2.Cal02329 were applied in a blastp analysis to identify proteins with high percentage of identity. Besides the obvious result for *C*. *saccharolyticus* strain DSM 8903, high percentage of identity was also found for endonucleases and methyltransferases from *C*. *naganoensis* strain DSM 8991 (> 97%), *C*. *changbaiensis* strain DSM 26941 (> 95%) and *C*. sp. strain F32 (> 99%) (S4 Fig). For each of the four strains, the genes encoding for the endonuclease and two methyltransferases are clustered within an operon identical to strain BluCon085 (S4 Fig).

To check whether the recognition sequence of the restriction endonuclease R.Cal02329 is methylated in other *Caldicellulosiruptor* species, genomic DNA of type strains of all twelve *Caldicellulosiruptor* species available at the Leibniz Institute (DSMZ–German Collection of Microorganisms and Cell Cultures GmbH) was digested with SfaNI. Our data show that genomic DNA of *C*. *saccharolyticus* strain DSM 8903, *C*. *changbaiensis* strain DSM 26941 and *C*. *naganoensis* strain DSM 8991 is not digested by SfaNI, whereas genomic DNA of all other *Caldicellulosiruptor* species is digested (Table 2). Unfortunately, genomic DNA of *C*. sp. strain F32 could not be included in the experiment.

## Discussion

The presence of restriction-modification (R-M) systems in bacteria are a complicating factor in transformation experiments, and therefore several strategies to circumvent these systems have been considered [61–64]. In the genus *Caldicellulosiruptor*, it has been the deletion of the restriction endonuclease CbeI and the methylation of heterologous DNA with the corresponding methyltransferase M.CbeI that enabled transformation of *C*. *bescii* strain DSM 6725 and *C*. *hydrothermalis* strain DSM 18901 [30–32, 34]. The genetic methods developed for these strains have advanced the study of physiological processes [22, 65–67], and the improvement of lignocellulolytic activity [68–71] and ethanol production [9, 60] in *Caldicellulosiruptor*. To date, no reports on the transformation of other *Caldicellulosiruptor* species besides *C*. *bescii* and *C*. *hydrothermalis* are available in literature.

In the current study, a novel SfaNI-like R-M system was identified in *Caldicellulosiruptor* sp. strain DIB 104C, which is most closely related to the type strain *C*. *saccharolyticus* DSM 8903 [35]. The system consists of the restriction endonuclease R.Cal02329 and the methyltransferases M1.Cal02329 and M2.Cal02329 that recognize the reverse-complement sequences 5’-GATGC-3’ and 5’-GCATC-3’. Our data show that methylation of DNA with the identified methyltransferases reduces degradation by endogenous nucleases, which in its turn is sufficient to achieve successful transformation.

SfaNI-like R-M systems are Type IIS R-M systems that recognize asymmetric sequences and generally contain two methyltransferases, each methylating one of the two DNA strands. An SfaNI-like R-M system has been characterized before in *Enterococcus faecalis* NEB21 [72]. In this strain, M.SfaNI is encoded by a single gene, but amino acid sequence analysis of the protein showed that it contains two separate conserved domains, responsible for different DNA strand recognition and methylation.

Methylation of a DNA fragment with methyltransferases M1.Cal02329 and M2.Cal02329 could not entirely protect the fragment from degradation by endogenous nucleases present in a BluCon085 cell lysate. These data suggest that additional R-M systems must be active in the strain. In this regard, REBASE reports that about 90% of bacterial genomes contain at least one R-M system and about 80% contain multiple R-M systems. Furthermore, Vasu and Nagaraja [29] show a positive correlation between the size of a bacterial genome and the number of R-M systems encoded by it. When extrapolating the numbers to strain BluCon085 with a genome size of 2.6 Mbp, in total three R-M systems may be anticipated in the strain. In this regard, a tblastn analysis of the methyltransferase M.CbeI from *C*. *bescii* strain DSM 6725 against the whole-genome nucleotide sequence of strain BluCon085 did not identify a homologous methyltransferase in the latter strain. Together with the fact that genomic DNA of strain BluCon085 is digested by the restriction endonuclease HaeIII (Table 2), it is apparent that a homologous CbeI/M.CbeI R-M system is not among the anticipated additional systems.

Although methylation of heterologous DNA with the methyltransferases M1.Cal02329 and M2.Cal02329 allowed transformation of strain DIB 104C, the identification of other R-M systems and the consequent methylation of heterologous DNA with the corresponding methyltransferases may further increase transformation efficiency. Analysis of the Nanopore sequencing dataset and a follow-up restriction digestion experiment using the restriction endonucleases DpnI and SexAI showed methylation of the sequences 5’-GATC-3’ and 5’-ACCWGGT-3’ in the genomic DNA of strains DIB 104C and BluCon085 (Fig 2 and S5 Fig). This methylation pattern corresponds to the pattern of the *E*. *coli* K12 methyltransferases M.EcoKDam (5’-GATC-3’) and M.EcoKDcm (5’-CCWGG-3’), and therefore the plasmid pUC19-P_slp_-pyrE used for transformation of strain DIB 104C was isolated from an *E*. *coli* K12 *dam*^+^/*dcm*^+^ strain, in particular DH5a. Nevertheless, methylation by at least M.EcoKDam does not seem to be absolutely required, as transformation with the linear DNA fragment obtained by PCR, which contains two unmethylated 5’-GATC-3’ sequences, was proven to be successful.

A SfaNI-like R-M system similar to that identified in this study may be active in other *Caldicellulosiruptor* species too, including *C*. *saccharolyticus* strain DSM 8903, *C*. *naganoensis* strain DSM 8991, *C*. *changbaiensis* strain DSM 26941 and *C*. sp. strain F32. Improvement of these species and strains by genetic engineering has great value as some of these species show interesting characteristics for industrial application. For example, *C*. *changbaiensis* strain DSM 26941 and *C*. sp. strain F32 have been commended for showing unique lignocellulolytic activities [73, 74], while *C*. *saccharolyticus* strain DSM 8903 has been considered for hydrogen and biogas production [17, 75], ethanol production [35], and production of higher molecular weight compounds [76]. The latter strain also shows a broad range of growth substrates and sugar co-utilization capacity [77].

The identification of the SfaNI-like R-M system in *Caldicellulosiruptor* extents the genetic engineering toolbox that is currently available for this genus. In addition, the identification of the 114-bp deletion in the *pyrE* gene together with the possibility to transform linear DNA fragments are valuable learnings, as they facilitate the generation of *pyrE* mutant versions of promising strains for industrial valorisation. Among these strains is strain BluCon085 producing high concentrations of L-lactic acid. Genetic engineering brings along a fast route towards improving these strains for a more efficient biomass conversion and for a higher or more diversified product formation.

## Supporting information

S1 Fig Maps of plasmids pTrc-Ec_cal02329M1, pTrc-Ec_cal02329M2 and pUC19-P_slp_-pyrE. (JPG)

S2 Fig Nucleotide sequences from 5’ to 3’ of genes and constructs. (DOCX)

S3 Fig Digestion of genomic DNA of strain DIB 104C, strain BluCon085 and type strains of twelve *Caldicellulosiruptor* species with the restriction endonucleases HaeIII and SfaNI. Restriction digests were performed by incubating genomic DNA in the presence of the restriction endonucleases HaeIII (B) and SfaNI (C). A restriction digest without restriction endonuclease was included as control (A).
(PDF)

S4 Fig Blastp analysis of R.Cal02329, M1.Cal02329 and M2.Cal02329. The blastp analysis identified proteins with high percentage of identity in *C*. *saccharolyticus* strain DSM 8903, *C*. *naganoensis* strain DSM 8991, *C*. *changbaiensis* strain DSM 26941 and *C*. sp. strain F32. For each protein, the RefSeq accession number (with prefix ‘WP_’), the percentage of identity, and the name of the corresponding coding sequence are shown in this order.
(DOCX)

S5 Fig Digestion of genomic DNA of strain DIB 104C and strain BluCon085 with the restriction endonucleases DpnI and SexAI. A restriction digest without restriction endonuclease was included as control.
(PDF)

S1 Table List of primers used in this study. The names of the restriction endonucleases are given in brackets and the corresponding recognition sequences are indicated with an underline. Phosphorothioate nucleotide linkages are indicated with an asterisk.
(DOCX)

S1 Raw images (PDF)
