# Correction: A novel SfaNI-like restriction-modification system in *Caldicellulosiruptor* extents the genetic engineering toolbox for this genus

**DOI:** 10.1371/journal.pone.0321512

**Published:** 2025-04-01

**Authors:** Steve Swinnen, Christian Zurek, Marco Krämer, Rebecca M. Heger, Jan-Eike Domeyer, Jan Ziegler, Vitali A. Svetlitchnyi, Albrecht Läufer

The following information is missing from the Data Availability statement: The following sequences have in addition been submitted to GenBank: gene cal02329R (accession number OQ631004), gene cal02329M1 (OQ631005), gene cal02329M2 (OQ631006), gene cal02329M1 codon-optimized for expression in E. coli (OQ631007), gene cal02329M2 codon-optimized for expression in E. coli (OQ631008), fragment consisting of 5’ CIS1, Pslp-pyrE and 3’ CIS1 (OQ631009), and whole-genome of *Caldicellulosiruptor* sp. DIB 104C (CP116337).
