# Supplementary figures and images for: A novel SfaNI-like restriction-modification system in Caldicellulosiruptor extents the genetic engineering toolbox for this genus
Source: PLoS One. 2022 Dec 29;17(12):e0279562. doi: 10.1371/journal.pone.0279562 (PMC9799307; doi:10.1371/journal.pone.0279562)

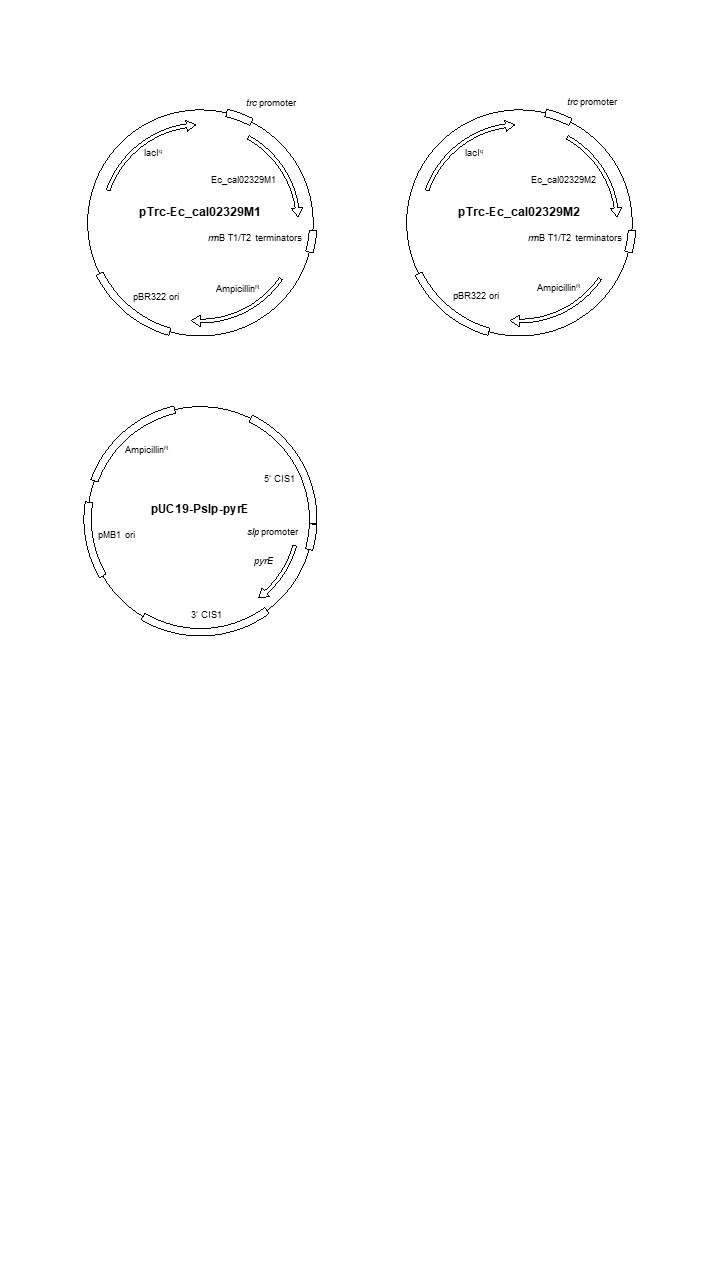

Supplement: S1 Fig — (JPG) [file pone.0279562.s001.jpg]

A

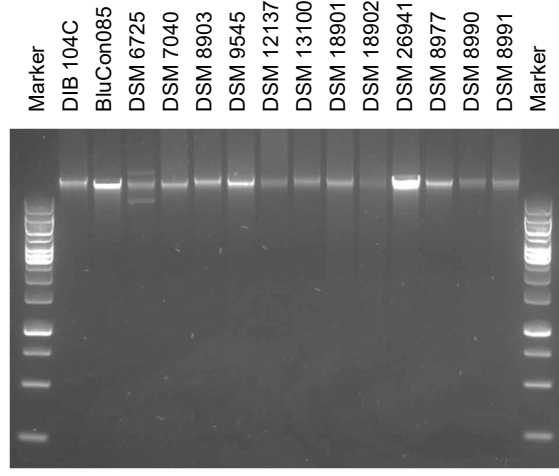

B

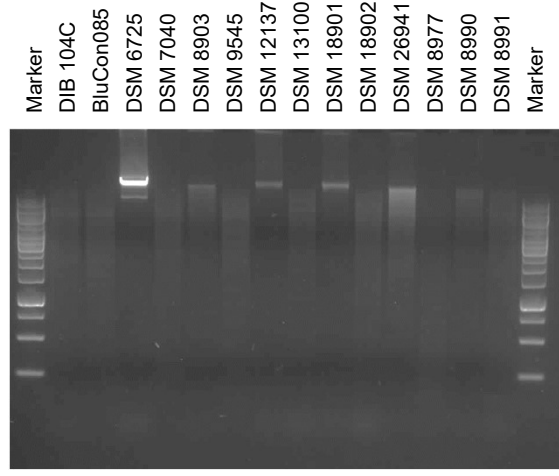

C

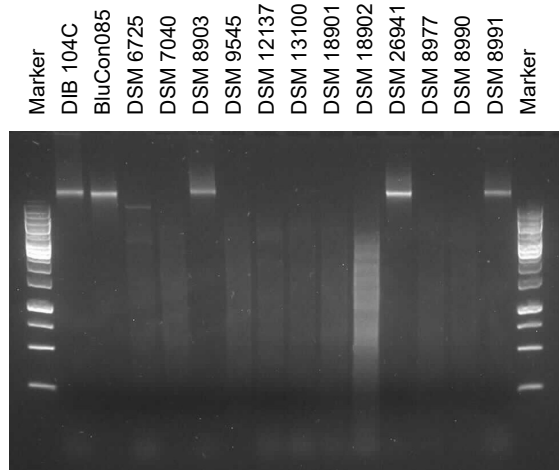

Supplement: S3 Fig — Restriction digests were performed by incubating genomic DNA in the presence of the restriction endonucleases HaeIII (B) and SfaNI (C). A restriction digest without restriction endonuclease was included as control (A). (PDF) [file pone.0279562.s003.pdf]

DIB 104C      BluCon085

Marker  
Control  
SexAI  
DpnI  
Marker  
Control  
SexAI  
DpnI  
Marker

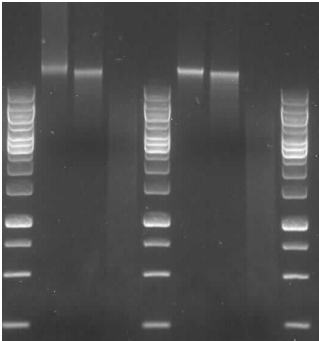

Supplement: S5 Fig — A restriction digest without restriction endonuclease was included as control. (PDF) [file pone.0279562.s005.pdf]
