# Supplementary material for: A novel SfaNI-like restriction-modification system in Caldicellulosiruptor extents the genetic engineering toolbox for this genus
Source: PLoS One. 2022 Dec 29;17(12):e0279562. doi: 10.1371/journal.pone.0279562 (PMC9799307; doi:10.1371/journal.pone.0279562)
Supplement: S2 Fig — (DOCX) [file pone.0279562.s002.docx]

***cal02329M1*, codon-optimized for expression in *E. coli***

| ATGATCTATAACACCATTCTGCATGGTGATTGCGTGACCATTATGAAAGAACATATTCCG  AGCGAAAGCATCGATCTGATTTATGCAGATCCGCCTTATAATCTGAGCGGTCGTGATCTG  ATTCTGAAAAATAACAAAACCGGTGGTCCGTTCTACAAGATGAATGAAGAATGGGATAGC  TGGGACTATGATAAATACTGCGAATTCACCTATAATTGGCTGCTGGCAAGCTATAGCGTG  CTGAAAAACAATGGTAGCCTGTATATTAGCTGCACCTATCATAATATTGGCGAGGTGATT  TTTCTGGCCAAAAAGATTGGTTTCAAACTGAACAATATTCTGACCTGGGTTAAAACCAAT  GCCATGCCGAATATTACCAAACGCACCTTTAAACACACCACCGAATTTGTTTGCTGGTTT  GTTAAAGGTCCTGGCTGGAAATTTAACTACAACGAGATCAAAATGCTGAACCCTCGCAAA  ACCAAAGATGGTAGCGTTAAACAAATGGACGATTTCTTCGACTTTTTTGAAATGCCGCTG  GTTCAGGGTAAAGAACGTATTAAACTGGATAATGGTCGTGCAGCACATCCGAATCAGAAA  CCGGAAAAACTGCTGGAAATCATTATTACCGCAAGCAGTGATGAAGGTGATATTGTTCTG  GATCCGTTTTTTGGCACCGGCACCACCGGTGTGGTTGCAGAACGTATGAATCGTAAATGG  ATTGGCATCGAAATCAACGAAACCTATATCGAGATTGCCAAAAAGCGCATTGAAGAGGAA  CGTCGTAAAAATGTTCAGAGCACCTTTATCTAA |
| --- |

***cal02329M2*, codon-optimized for expression in *E. coli***

| ATGTGCAAAGTGCACCTGTTTAATGATGATTGCCTGAACGTGCTGAAAAAGATCGAAGAT  AATAGCATCGATCTGATCTTTGCAGATCCGCCTTATAATCTGAGCAGCGAAAATGCACTG  ACCACACGTGCAGGTAAACCGGTTAAATGTTATAAAGGCGAGTGGGATAAAATCGACGAT  ATCTTTGAATTTAACCTGCGCTGGATTGAACAGTGTGTTCGTGTTCTGAAAGAAACCGGC  ACCATTTGGATTAGCGGCACCCTGCATAATCATCCGATTATTGGCACCATTCTGAAACAG  TTAGGTCTGTGGATTATCAACGATATCATTTGGTTCAAACCGAATGCAACACCGCTGCTG  AGCCGTAATCGTTTTGTTCCGAGCACCGAACTGATTTGGGTTGCAAGCAAAAACAAAAAA  TACTACTTTGATTATGAAATGGCACGCAAACTGAATGGTGGTAAACAAATGCGTAATCTG  TGGGAAATTCCGGCACAGCGTCATAAAACACCGCATCCGACCGAAAAACCGGAAGCACTG  CTGGAACGTATTATTCTGATTGGTAGCAAAGAAGGTGACGTTGTTCTGGATCCGTTTATG  GGTAGCGGTACAACCGGTGTTGTTGCAAAACTGCTGAAACGCAACTTTATTGGCATTGAA  ATTGATCCGGTGTATTTCGAGATTGCCAAAAAACGCATCGAAGAAGAAAAACTGATTCAG  CAGACCTTTAGCAACTTCCTGTAA |
| --- |

**Fragment consisting of 5’ CIS1, P_slp_-*pyrE* and 3’ CIS1**

| ATCAGTCTTACTATCTGCCTTGATTATTCCAAAATATGGCATGACGGTTAGAAAATAAAT  TTCCTTTCCCCAAACGACTTTTCAAAATATCTCTGCAAAAAGTATTTATCACATCCTGTT  TTTGTACTTTTGGTTTCTTGGAGTTTATTGTTAGTCGGGTTACGAATTGAAGATTGTTTT  AAAAGGTCCCTGAAGTTTGGTGAAAGAAATTATATAATCATATAATAAAACCTGGCAAAA  AAAAAAAAAAAAAAAAAAAAAACATTATATGGAATTTAATGGAAAAACTAACCTTCTGGC  AAATGTGAAGGGCAGAAAAAGAAGGCAAGGAGGTTTTTGTTTTCTACATGCTGCAAACAT  TTGAAAAGTAATAATTATGTGCTTTGTTGTTAGCCTTAAATGCACAAATGCAAGCTATTC  AAGGCAATATTTGACTTGAAAAAAGGCAGCTGGTAAAGATTAATGCCAGGTGCTTTTTGT  TTTATACAAAAAATTTTTAAAATTGTTTAACCATCAATGTGGAAGGCTAATTTTTTTATG  TTAGATTTCATTTATTTTATTTGTTTTTTTCACAATAGCTTTATTGTTTTTTATAAAGAT  TTAACCTAAAGTTTTTACTTCAATTTTAGAATTGTTTTTAATTATTGGTTCTACTTTGTA  TTGGGGATTACCAGGTAGATTAGTATCCTATATATATTCTAAGGCTCAATGGAGTCATGA  TTTATTAAGCTTTTGTTAAGGTGGGAGGAGCAGTTATGTATGATGTGGTCGAAGAGAAAA  TCAAACAATATGAAAAATTGTTAATTGAAATGTGGACTGCATCTGAGAAGTATCTTGGCA  GAACTTCTGTCCAGCTTTTAGTAGAAAGAGTTGTATGGGAACTTTCAGCAGAATACAAGG  AAATAGAATAACTTTACTTTGATGAAAATGGAATATCATGTAAAGAGATATTAACAGTTT  TAAAGAAAAACCCAGAATTTCCCGTAGACAGGAATTCTATGCAGATATTCAAGGTTTAAA  AAGTTTTTTGGGCTTTTGCAAAATTATTTCACCAATTTTACAGTTTGGTTACAAATGCTA  AAGACCTATTGACTTATTAAAATGCTGTTGTTAAAATTTTAGCTGTAAGTGATGAGGCTA  TAAAAAAGCAAGAGCCTCATCACTAAAAAATCATACAAGGAGGTTTGGTGAGTAGTTATG  AATAAAGAGGCTTACATTCAAATGTTCAAAGACACAGATGCACTTTTGGAAGGACATTTT  CTTTTGTCCTCTGGAAAACACAGTGCAAAGTACCTTCAATGTGCAAAAGTGTTGCAGTAC  CCAAACTTGGCAGAAATGATCTGCAGGGACCTTGCACAATACTTTAAAGATAAGCAAATT  GACGTTGTTATAGGCCCTGCGTTGGGAGCAGTAACGCTTTCGTACGAACTTGCAAGACAG  TTAAATTGCCGTTCCATCTTTGCAGAAAGAGAAGATGGGATAATGAAACTTAGAAGAGGA  TTTAAGATTGAAGAGGGAGAAAAAGTTTTGGTAGTTGAAGACGTCATAACAACAGGCGGG  TCTGTGAAAGAAATAATTGAAATTGTAAAAGAGTACAAAGGAGAAATTGTGGCAGTTGCT  GGCATTGTAGATAGAAGTGGTGGAAAGGTAGAACTTGGCTATCCTTTGAAAACTCTTCTT  ACACTTGAGATTGAAACATATGAGCCTGAAGAGTGTCCGCTTTGTAAAGAAGGTATACCT  ATTGTAAAACCTGGAAGTAGAAAAAGTAAGTAGGTCGACTTACGTTGAGATTCTGAGTAG  GTTGATTGGTAGTGAAAATGCAGGTAAGCTCATGGAAATACTACGCCAGGAAATGAATGA  GGTGTATTTTGATTAGGGAGGGCAAAGAAGATGGAAAGATTGAAAACAGGTATTAAAAAT  CTTGACCGTGTTCTTGGTGGGGGCATACCGCTGTATTCGCTGAATATTGTATCTGGTGCG  CCAGGCAGTGGAAAAACAATATTTGTTCAAAATATAGTGTTTAACAGTGCAAGAAATGGA  CTTAAGAGTTTATACTTGACAACTATTTCAGAATCACAATTTAAGATGGTAAGACATTTA  CAAGAATTTAAGTTTTTTCTGACGATTTACTTGGCGGCAAGTTTATTTATGCAGACCTTG  GAGAAGTTGTGCGCAAACAAGGACCTAGTAAGATTTTAGGGTACTTGACTGACATGGTAA  AAAAGTACAAGCCTAACATAATTGTGATTGACAGCTTTAAAGCTATAAGAGACATATTCC  CTGACGAAAAGACTTTTTAGGCTTTTGTTTTTGATCTGGCTGCTGCATTGTCAATATGGG  AAGTTACTGTGTTTCTTATAGGTGAGTATGAAGAAAAAGAATTAACTGTCTTGAGTGAAT  TTGCGATTGCGGATGGGACTTTTCATCTTTATGGACAGGAGGAAAAGAAGTTCCAGAAAA  GATATTTGCGTATCTTAAAGATGAGGGGGACTTCTTTTGAACAGGGCGAGCATTTGTTTG  AGATTACTCCTGCAGGGATAAAAGTGTATCCCAGAATAAAACCGGCTGGAGAAGAGCTTC  AGTATGAAGTCAAAGCGGAGAAAAAAGGATTTGGAATCAGAGATTTGGACGAGATGCTAA  ATGGTGGCCTACCAGAAGGAACTATTACTATCATCTCAGGTGGGACTGATACAGGCAAGA  CCACGCTTGCTCTTAAATTCTTACTTGAGGGGGCTGAGATAGGCGAGAAGGGATTATTGC  TTTCTTTTGAGGAACCTCTTGCTTAGCTTA |
| --- |

***cal02329R***

| ATGCGAAAGCCTTGGTCTATTTCCACAACTGTTAGAAACCCCGAAAGATTAAGAGGATTT  TTGCAAGTGTTATCTGAGTTTGAAGGAATGAATTTTGATGAGAACGTTCAAATTCAATAT  CAGATACGATTAATACAATACAAACTCTACAGACCTATGAATTTACCTGAAGATATAAAA  CGTGAGTTTGATGACCCCGCAATTAAAAAAATAGATTACAAAAAAGCTAAGAAGATTTTT  GATTTACAAAAATATAAAGATCCTTCTATGCGTGGTCGGCAGTCTGTAAATCCCTTGAAC  AAATTAGGATTTGCAATTGCCAAAAAAAGCTTAGGCAAGATTAGAATAACTGAGTTGGGA  AGAAAATTTTTAGATGAAAATAACGATGTCTCCGAGATACTTTTCAAAAGTCTTCTTAAA  TTACAGTACCCAAATCCTTTTAGTAGCGATTTTAAAAGCAGAGATGGCTTTGATATCATT  CCTTTTATAGTAACTTTGAAGTTTTTCTATTTGTTAGAAAAACAAACGCAAATAGAAGAA  ATTTCAAAAAGAGAATTTTGTTTATTCATTCCAACCCTCATTAACTATAAAGAAATAGAA  AATCAAATTAACCAGCTACTTGATTATAGAAGATCAAAAAATAAGAGAGATTTTGAAGTA  CATTTCGTCTCTAAGTTTTTTGGTTCAGCTAAAAATATTGAGACTAAAATCAACAATTTG  TTTGATTATGGAGATAACATCTTACGATTTTTTCTTCTTACAAAATGTTTTACCGCTAAA  AAAAGTGAATTTGGACAAGTTGCCAGTGTAAAACTTTCACAAGATAGAAAAAAGGAAATC  GAAGAATTACTAAATATGTTTGAAGGAAAAGCAATAAGCTTTTCTAATTTAGATGACTAC  ATCGAATATATGACTGATATCACAAAACCTGAATTGCCGTGGGAAAAAAATAAGAATAAA  CTTATTGAGATGGCAGAAAGTATCCGGAAAGATATTTCACAGCAAATAGAAAGCAGTAAA  ATTGCAATAAATGAATACTCTAAAATGGTTTTAGGAAAAAACTTATTTGAGTTATCTGAG  GAAGAATTGAAAAAACATATCAATGAACTGAGGGCGATAAAATCCAAAATTAACGAAGCT  AAAAAGGCTCATATCTTAAAATATAACTTTGCTAAATTAGACGAACATATTAAAATACTA  AGGAATAAAGAATATTGGAAGGAATTAGAACCAGCCGATTTAGAACAAATAATATTCGAG  CTATTGTTAATTATTGATAGTGCAGAAAAAATTGAATCAAATGCTATTAAAGATGATGAA  GGAAATTTTATAAATTTTGCACCAGCTAAAAAACCTGATATTGAATTTTTCTTCAAGGAA  TTTGCTGGAATTTGTGAAGTAACACTAAACAAAACTCAATATCAATGGATTCAAGAGGGA  TATCCTGTATTAGATCACGTGGCAAAATTTATGAATCAATACCCTAATTATACTAATTTT  GTAAATATTTTTATTGCTCCAAAAATACATGATAATACCTATTATAATTTTTTCATTGCT  TTAAAATACGGATTTAAGAGTAAGAAAATCAGAATTATCCCTTTAAATTTTGAACAGTTT  ACTATGTTCACAAAAGTGCTTCAAAGCTATTTTGAAAAGTTCAATGGTTTAAATTCAAAC  TTAATTTTGACCCTTTGTAATGATATATTTTACACAATGGAAAATTTAGATGACCACTCA  ATGATATGTAGTTTAATTGACAATAAGCTTTATAGTCTATTTTAG |
| --- |

***cal02329M1***

| TTGATTTACAATACAATTTTACATGGTGATTGTGTAACGATTATGAAAGAACATATTCCA  TCGGAAAGCATAGATTTAATTTATGCTGACCCACCTTACAATTTGTCAGGCAGAGATCTT  ATACTAAAAAATAACAAGACTGGTGGTCCATTTTATAAAATGAATGAAGAATGGGACAGC  TGGGATTATGACAAATACTGTGAATTCACTTATAATTGGCTTTTAGCCTCATATTCTGTC  TTGAAAAATAATGGTAGTTTGTATATTTCTTGTACTTACCATAACATTGGAGAAGTTATA  TTTTTAGCAAAAAAGATAGGCTTTAAATTAAACAATATATTGACATGGGTCAAGACAAAT  GCTATGCCAAATATTACTAAACGTACATTTAAACACACAACAGAATTTGTTTGTTGGTTT  GTTAAAGGCCCTGGATGGAAATTTAACTATAATGAAATTAAAATGCTTAATCCAAGAAAA  ACAAAAGATGGCTCTGTTAAGCAAATGGATGACTTTTTTGATTTCTTTGAAATGCCTCTT  GTTCAAGGAAAAGAAAGAATTAAGTTAGACAATGGCAGAGCCGCACATCCAAATCAAAAA  CCTGAAAAATTGTTGGAAATAATAATTACCGCTTCAAGTGATGAAGGAGATATAGTATTA  GATCCTTTTTTTGGAACAGGAACAACTGGTGTTGTTGCTGAGCGTATGAATAGAAAATGG  ATTGGAATTGAAATAAACGAAACCTACATTGAAATTGCCAAAAAGAGAATTGAAGAGGAG  AGAAGAAAAAATGTGCAAAGTACATTTATTTAA |
| --- |

***cal02329M2***

| ATGTGCAAAGTACATTTATTTAATGATGATTGTTTGAATGTTCTAAAAAAGATAGAAGAC  AATAGTATAGATCTGATTTTTGCTGATCCTCCTTACAATTTGTCTTCAGAAAATGCTCTA  ACCACGAGAGCCGGTAAACCAGTAAAATGTTATAAAGGCGAATGGGATAAAATAGATGAT  ATATTTGAGTTTAATCTAAGGTGGATCGAGCAATGTGTCAGAGTACTTAAAGAAACTGGA  ACTATTTGGATTTCTGGAACATTGCATAATCATCCTATAATTGGAACTATTCTGAAGCAG  TTGGGTCTCTGGATTATCAATGACATTATATGGTTTAAACCTAATGCAACTCCTTTACTT  TCAAGAAATAGATTTGTTCCATCTACAGAATTGATCTGGGTTGCAAGTAAAAATAAAAAA  TATTATTTTGACTATGAGATGGCACGAAAGCTTAATGGAGGCAAACAGATGAGAAACTTA  TGGGAAATTCCTGCTCAAAGGCATAAGACTCCTCATCCTACTGAAAAACCTGAAGCATTG  TTAGAAAGGATTATTCTAATAGGCAGTAAAGAAGGGGATGTGGTCTTAGATCCTTTTATG  GGCTCTGGAACAACTGGCGTTGTAGCTAAATTGCTTAAACGAAACTTTATTGGAATTGAA  ATTGATCCAGTATACTTTGAGATTGCAAAAAAACGTATTGAGGAAGAAAAGCTTATTCAG  CAAACTTTTTCAAATTTTCTTTAA |
| --- |
