# Supplementary material for: A novel SfaNI-like restriction-modification system in Caldicellulosiruptor extents the genetic engineering toolbox for this genus
Source: PLoS One. 2022 Dec 29;17(12):e0279562. doi: 10.1371/journal.pone.0279562 (PMC9799307; doi:10.1371/journal.pone.0279562)
Supplement: S4 Fig — The blastp analysis identified proteins with high percentage of identity in C. saccharolyticus strain DSM 8903, C. naganoensis strain DSM 8991, C. changbaiensis strain DSM 26941 and C. sp. strain F32. For each protein, the RefSeq accession number (with prefix ‘WP_’), the percentage of identity, and the name of the corresponding coding sequence are shown in this order. (DOCX) [file pone.0279562.s004.docx]

| ***C.* sp. DIB 104C** | R.Cal02329  M2.Cal02329 | M1.Cal02329 |  |
| --- | --- | --- | --- |
| ***C. saccharolyticus* DSM 8903** | WP_011918235  97.5% CSAC_RS13975 | WP_011918236  97.8% CSAC_RS13980 | WP_011918237  98.8% CSAC_RS13985 |
| ***C. naganoensis* NA10** | WP_045166046  97.8%  N907_RS09635 | WP_200889487  99.3%  N907_RS09630 | WP_045166045  99.2%  N907_RS09625 |
| ***C changbaiensis* DSM 26941** | WP_127352869  95.0%  ELD05_13760 | WP_206516897  99.5%  ELD05_13765 | WP_127352870  97.6%  ELD05_13770 |
| ***C.* sp. F32** | WP_039765893  99.7%  H964_RS06575 | WP_039765895  99.6%  H964_RS06580 | WP_039765897  100.0%  H964_RS06585 |
