# Supplementary material for: A novel SfaNI-like restriction-modification system in Caldicellulosiruptor extents the genetic engineering toolbox for this genus
Source: PLoS One. 2022 Dec 29;17(12):e0279562. doi: 10.1371/journal.pone.0279562 (PMC9799307; doi:10.1371/journal.pone.0279562)
Supplement: S1 Table — The names of the restriction endonucleases are given in brackets and the corresponding recognition sequences are indicated with an underline. Phosphorothioate nucleotide linkages are indicated with an asterisk. (DOCX) [file pone.0279562.s006.docx]

| **Primer name** | **Primer sequence from 5’ to 3’** |
| --- | --- |
| BLU01 | ATGACGGTGAAAACCTCTGAC |
| BLU02 | GACTTGGTTGAGTACTCACC |
| BLU03 | AAAAGGATCCATCAGTCTTACTATCTGCCTTG (BamHI) |
| BLU04 | AAAAGAATTCCTGTCTACGGGAAATTCTGG (EcoRI) |
| BLU05 | AAAAGTCGACTTACGTTGAGATTCTGAGTAGG (SalI) |
| BLU06 | AAAATCTAGATAAGCTAAGCAAGAGGTTCC (XbaI) |
| BLU07 | AAAAGAATTCTATGCAGATATTCAAGGTTTAAAAAG (EcoRI) |
| BLU08 | GAACATTTGAATGTAAGCCTCTTTATTCATAACTACTCACCAAACCTCC |
| BLU09 | ATGAATAAAGAGGCTTACATTCAAATG |
| BLU10 | AAAAGTCGACCTACTTACTTTTTCTACTTCCAGG (SalI) |
| BLU11 | GTTGCTCCTCATGTTTCTGC |
| BLU12 | TATCCATTTCCCATCCCGAG |
| BLU13 | ATCAAGACTGGTGGCAACAG |
| BLU14 | GCACTTCCAACATTCTGTCC |
| BLU15 | A*G*G*TGGACTTTCAGGCCCTGCTATAAAGCC |
| BLU16 | A*A*C*TTGCAGGGACACTTTCTGGCGGAGAAC |
| BLU17 | GTACGAACTTGCAAGACAG |
| BLU18 | GCACTTCCAACATTCTGTCC |
