# Supplementary material for: A novel SfaNI-like restriction-modification system in Caldicellulosiruptor extents the genetic engineering toolbox for this genus
Source: PLoS One. 2022 Dec 29;17(12):e0279562. doi: 10.1371/journal.pone.0279562 (PMC9799307; doi:10.1371/journal.pone.0279562)
Supplement: S1 Raw images — (PDF) [file pone.0279562.s007.pdf]

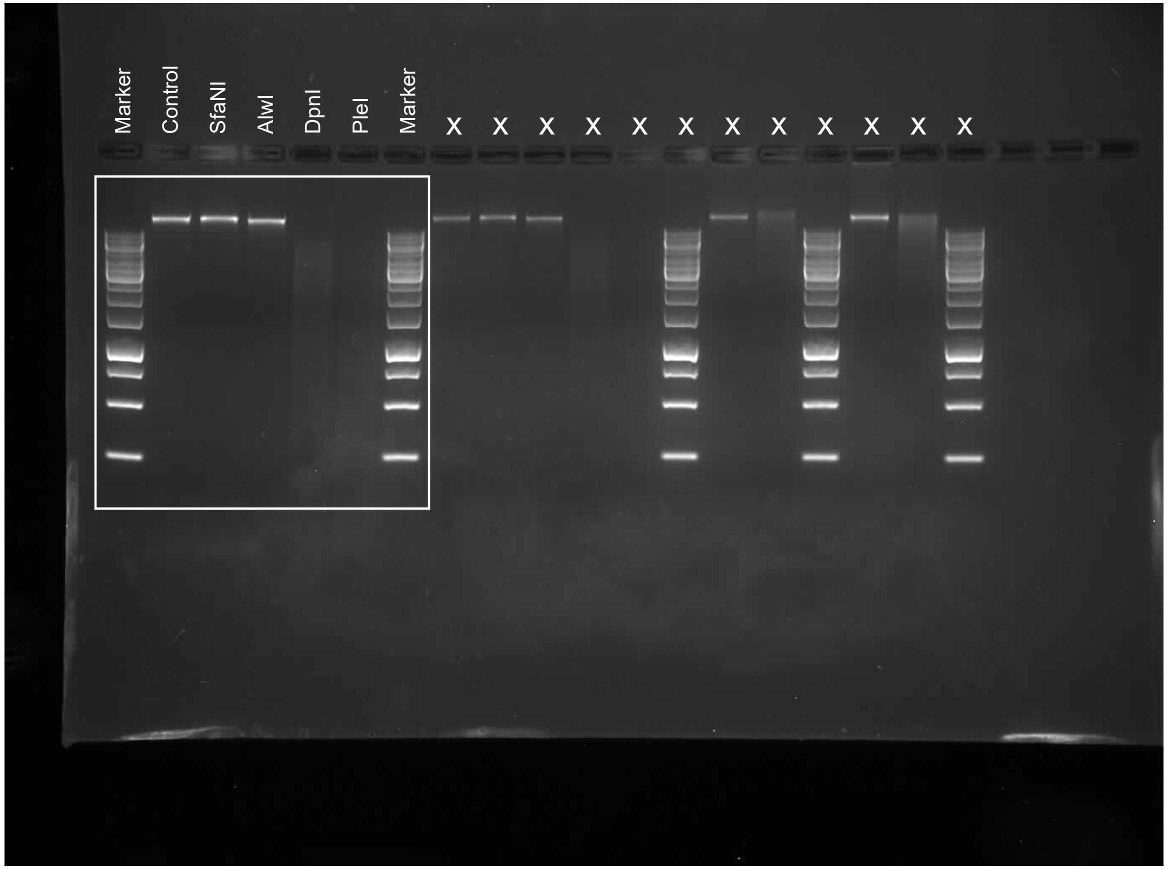

The image was captured with VisionCapt software from Vilber (exposure time of 1.6 sec)

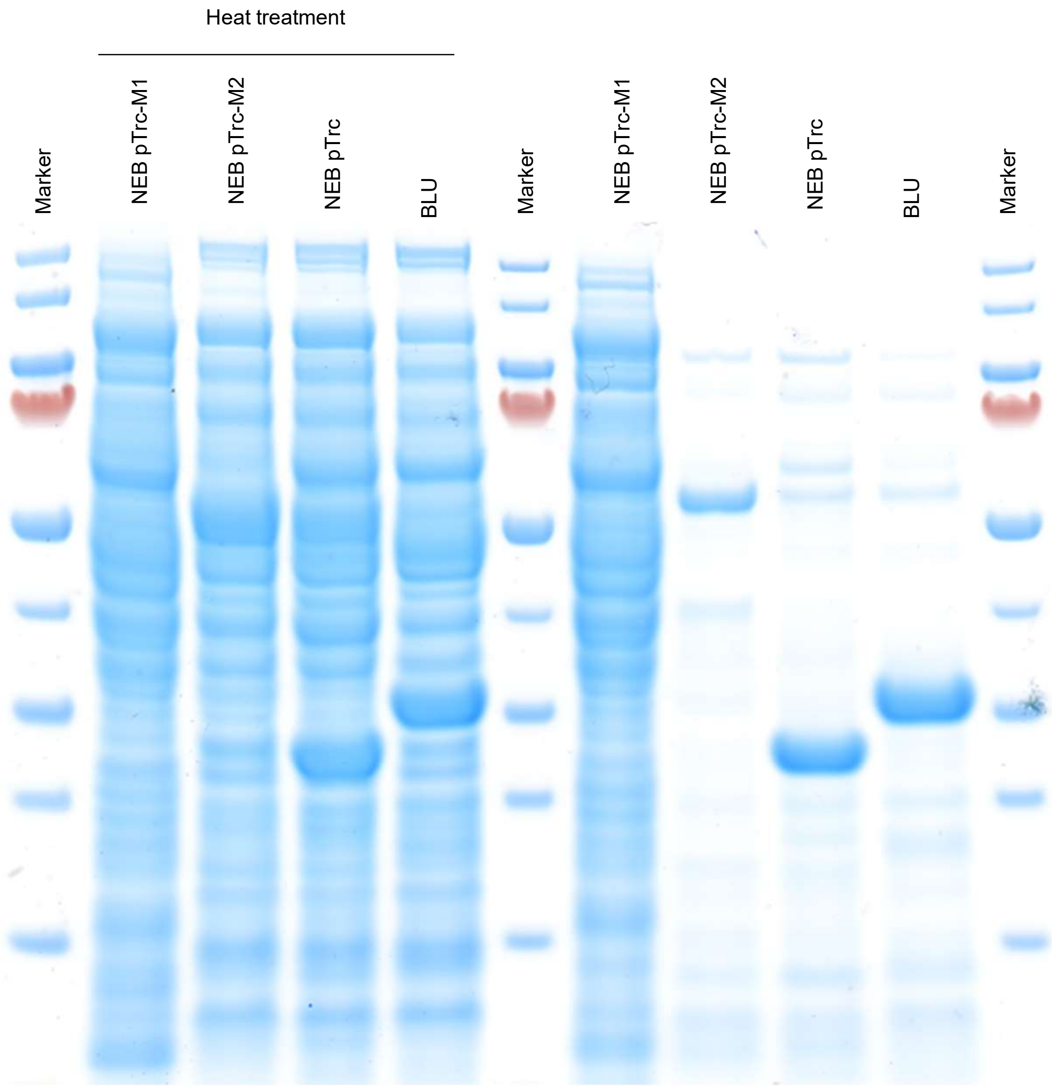
Heat treatment
Marker
NEB pTrc-M1
NEB pTrc-M2
NEB pTrc
BLU
Marker
NEB pTrc-M1
NEB pTrc-M2
NEB pTrc
BLU
Marker

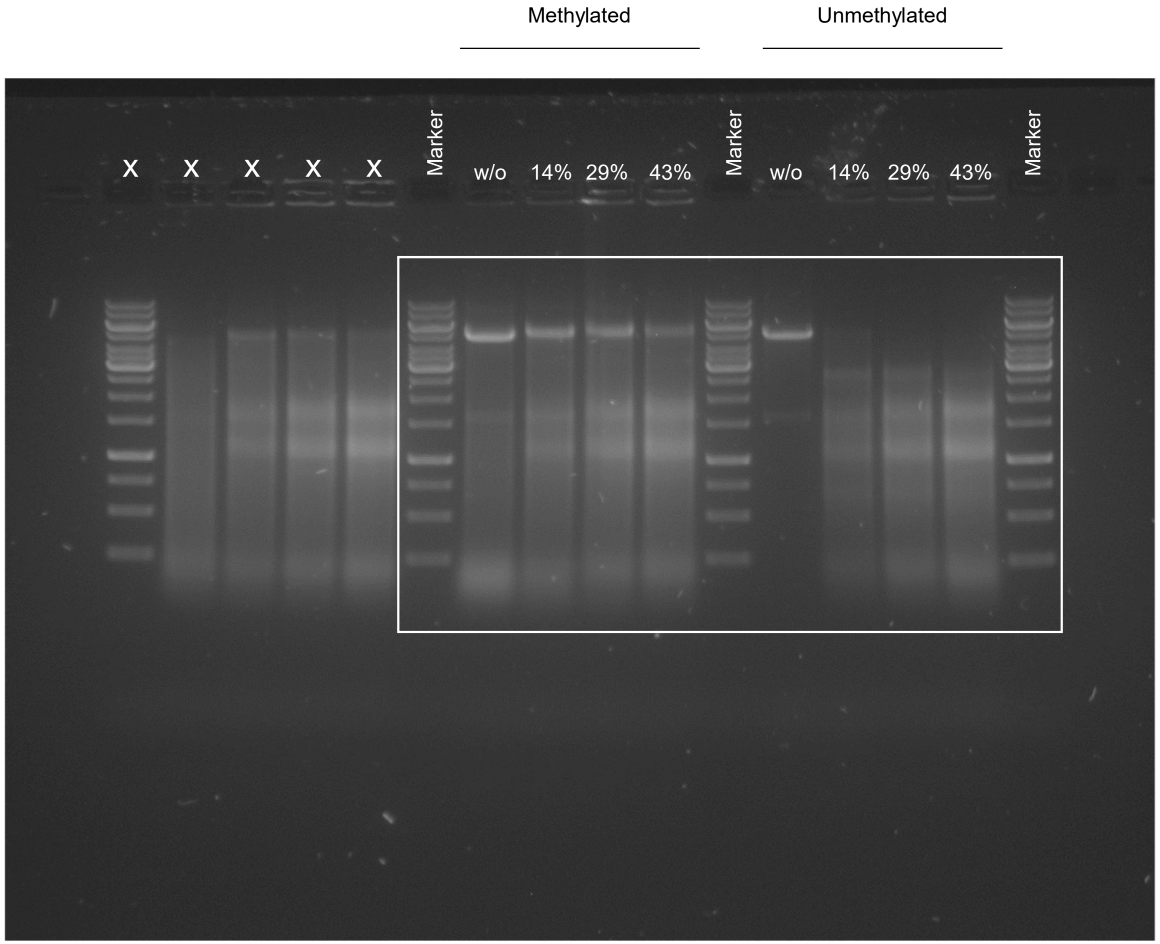

The image was captured with VisionCapt software from Vilber (exposure time of 1.6 sec)

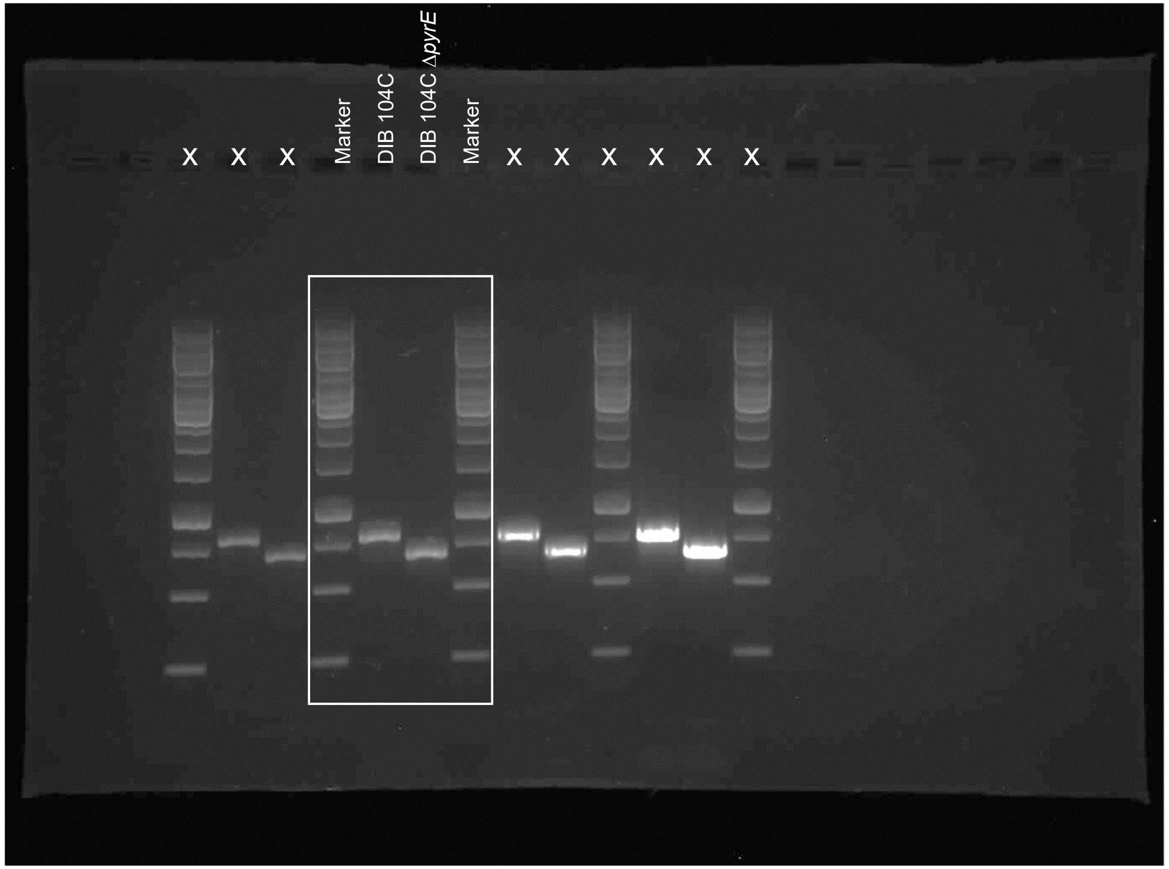

The image was captured with VisionCapt software from Vilber (exposure time of 1.6 sec)

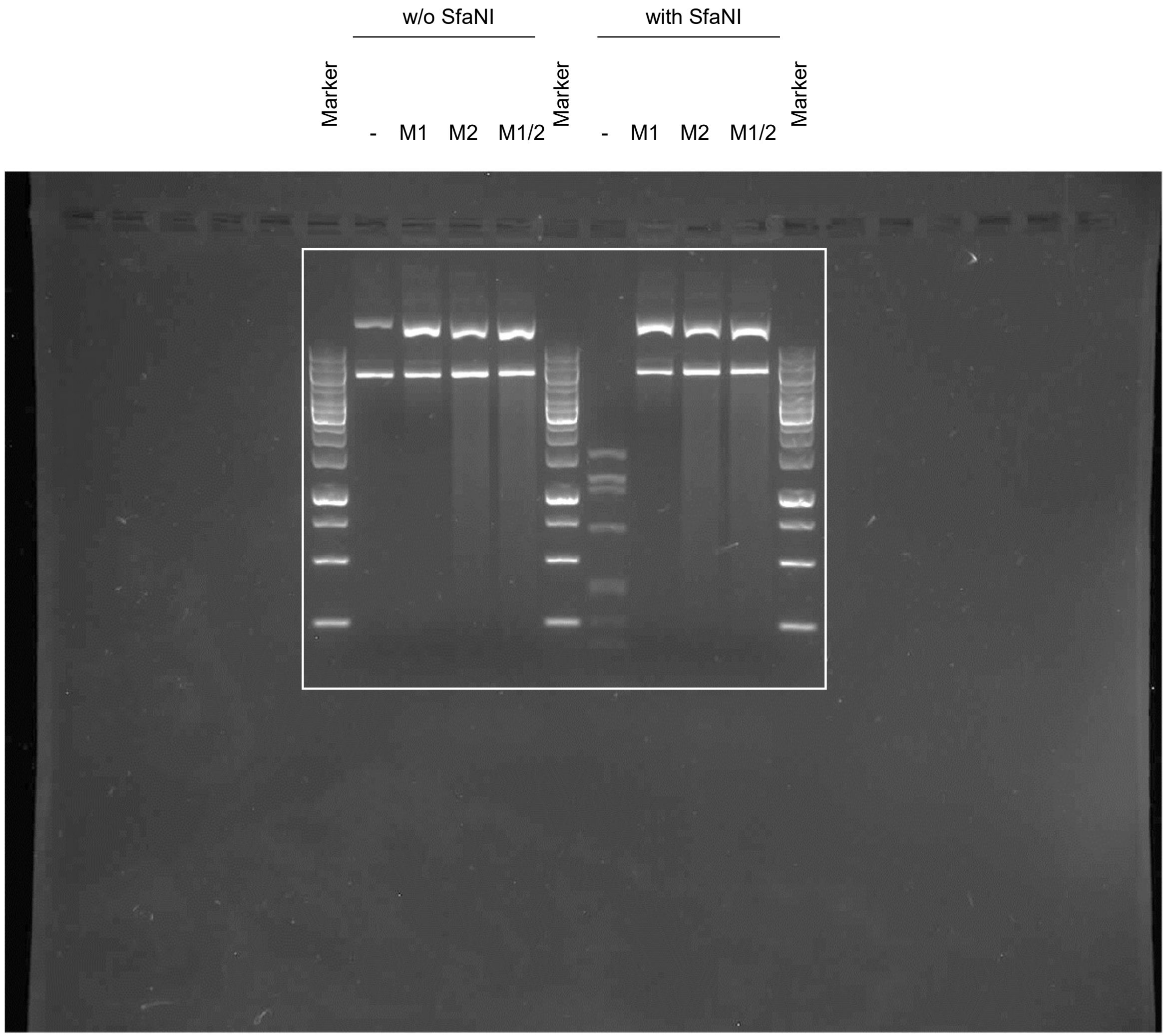

The image was captured with VisionCapt software from Vilber (exposure time of 1.6 sec)

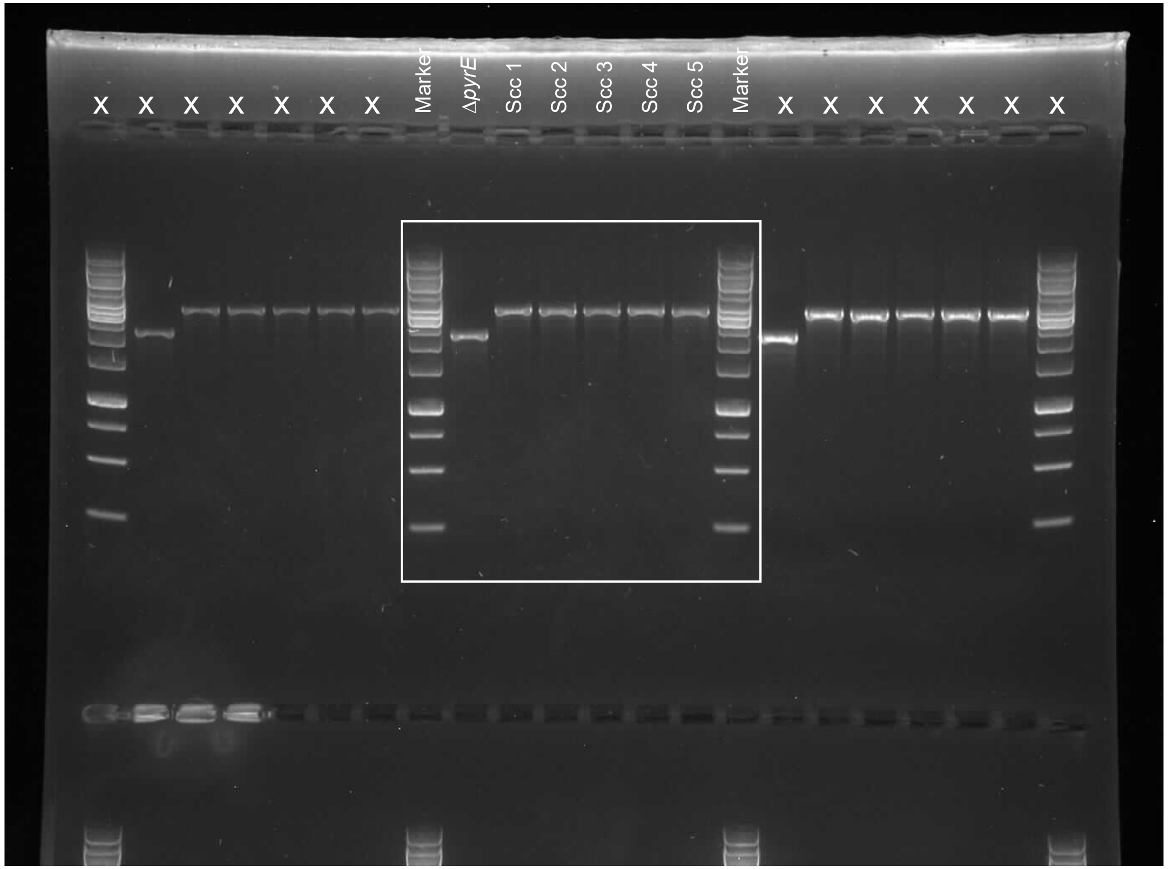

The image was captured with VisionCapt software from Vilber (exposure time of 1.6 sec)

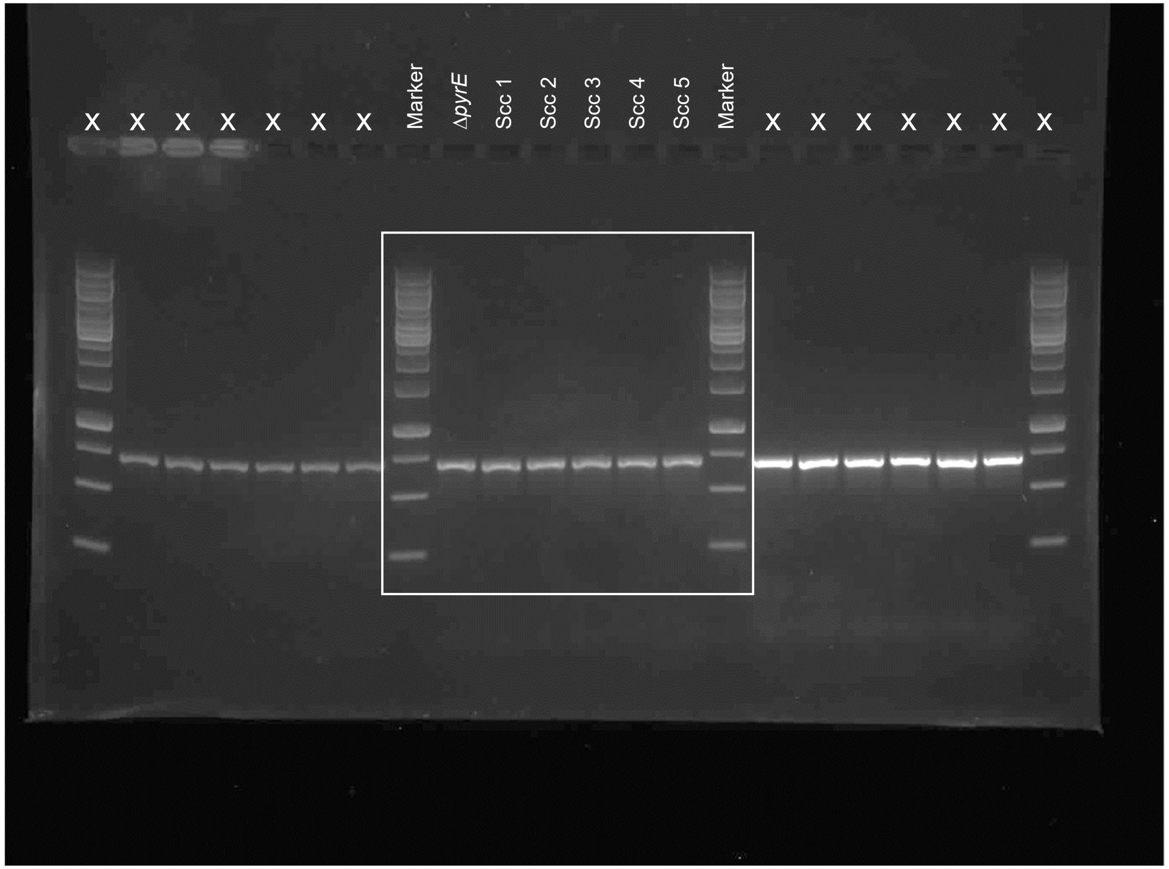

The image was captured with VisionCapt software from Vilber (exposure time of 1.6 sec)

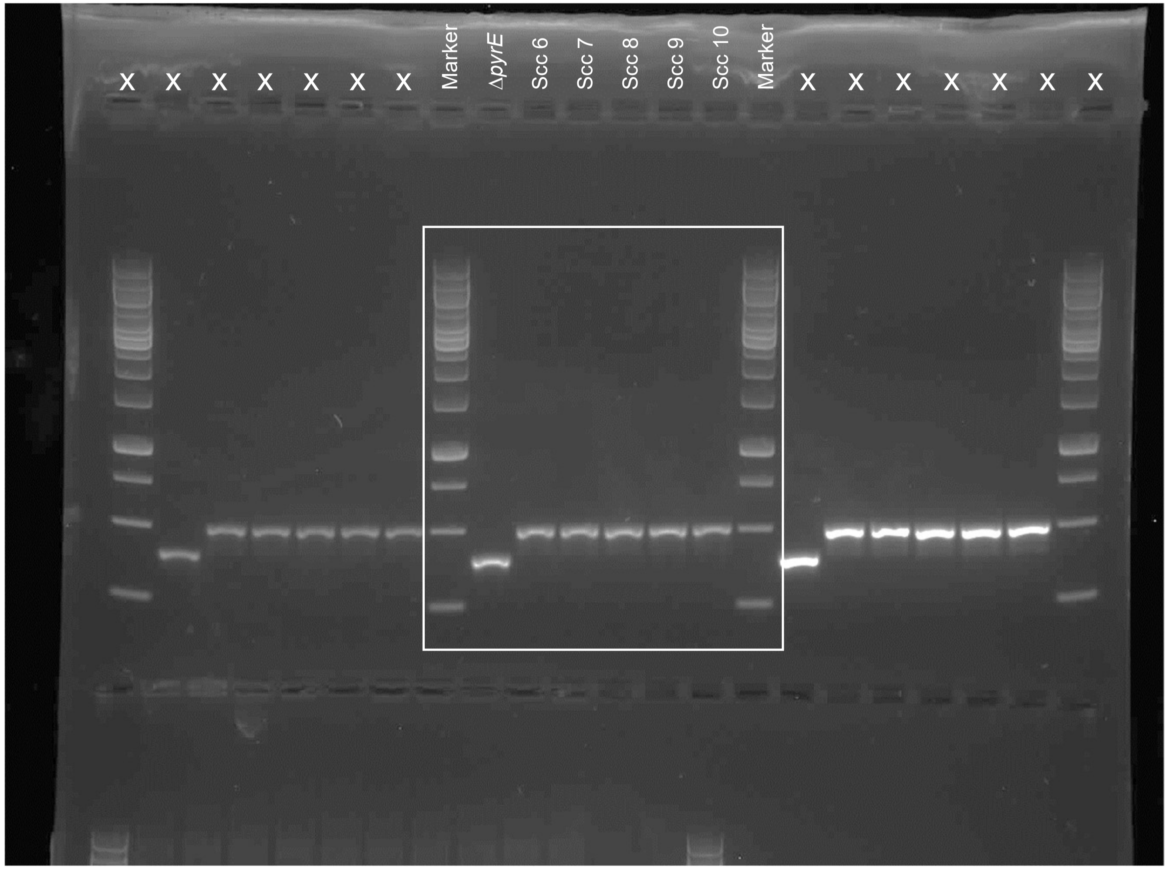

The image was captured with VisionCapt software from Vilber (exposure time of 1.6 sec)

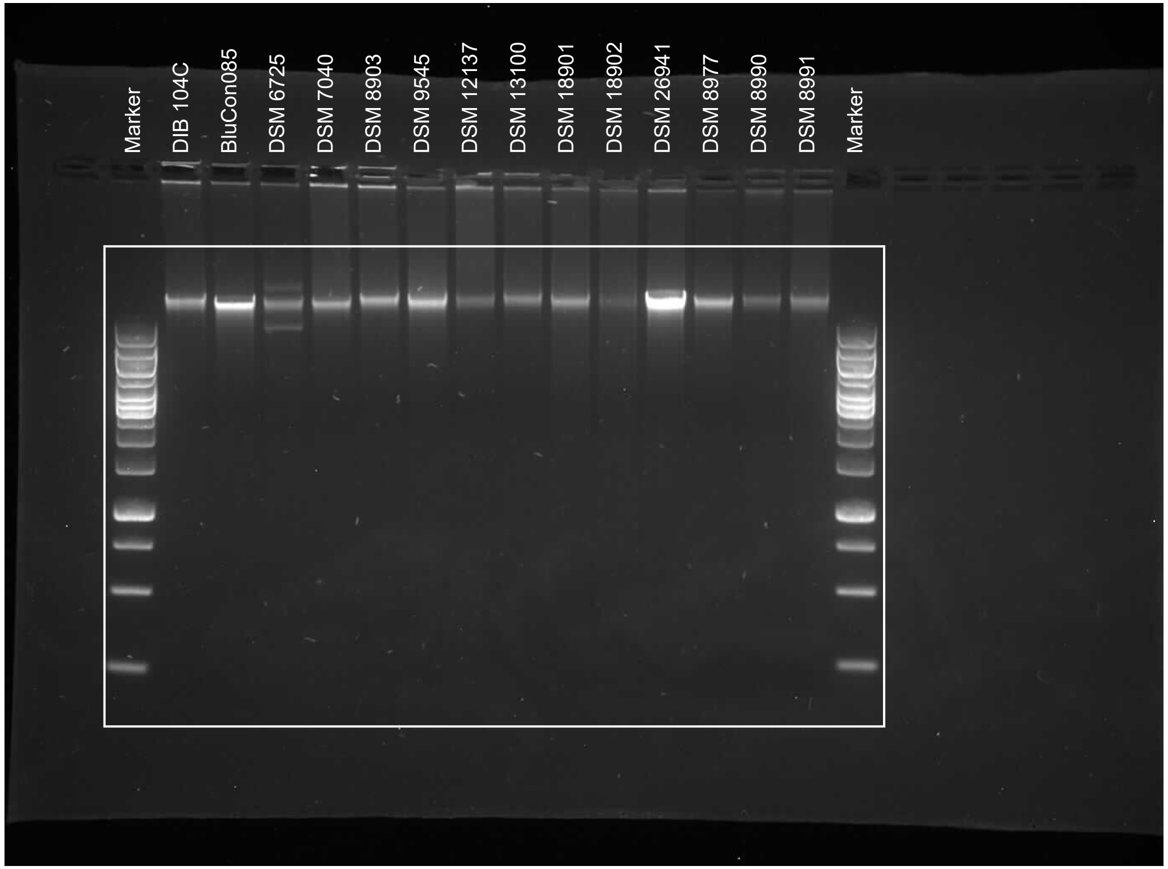

The image was captured with VisionCapt software from Vilber (exposure time of 1.6 sec)

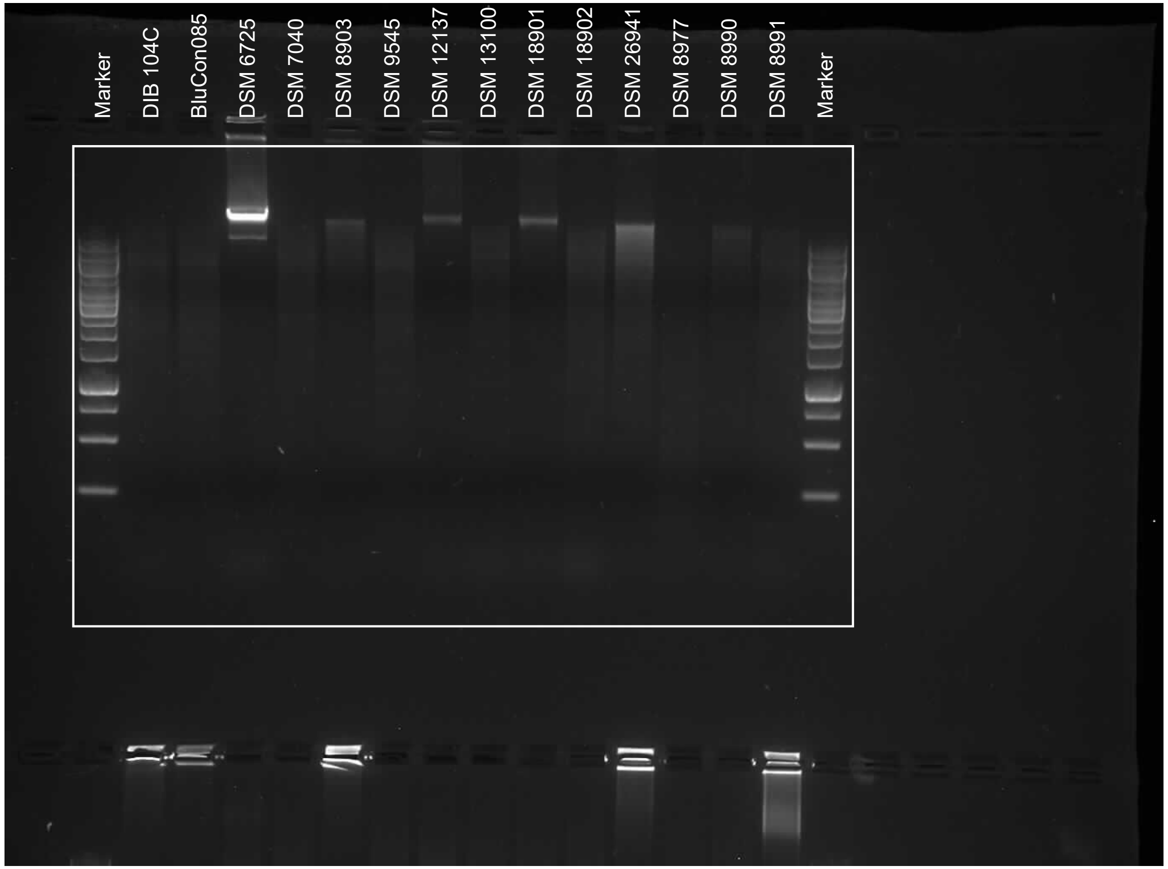

The image was captured with VisionCapt software from Vilber (exposure time of 1.6 sec)

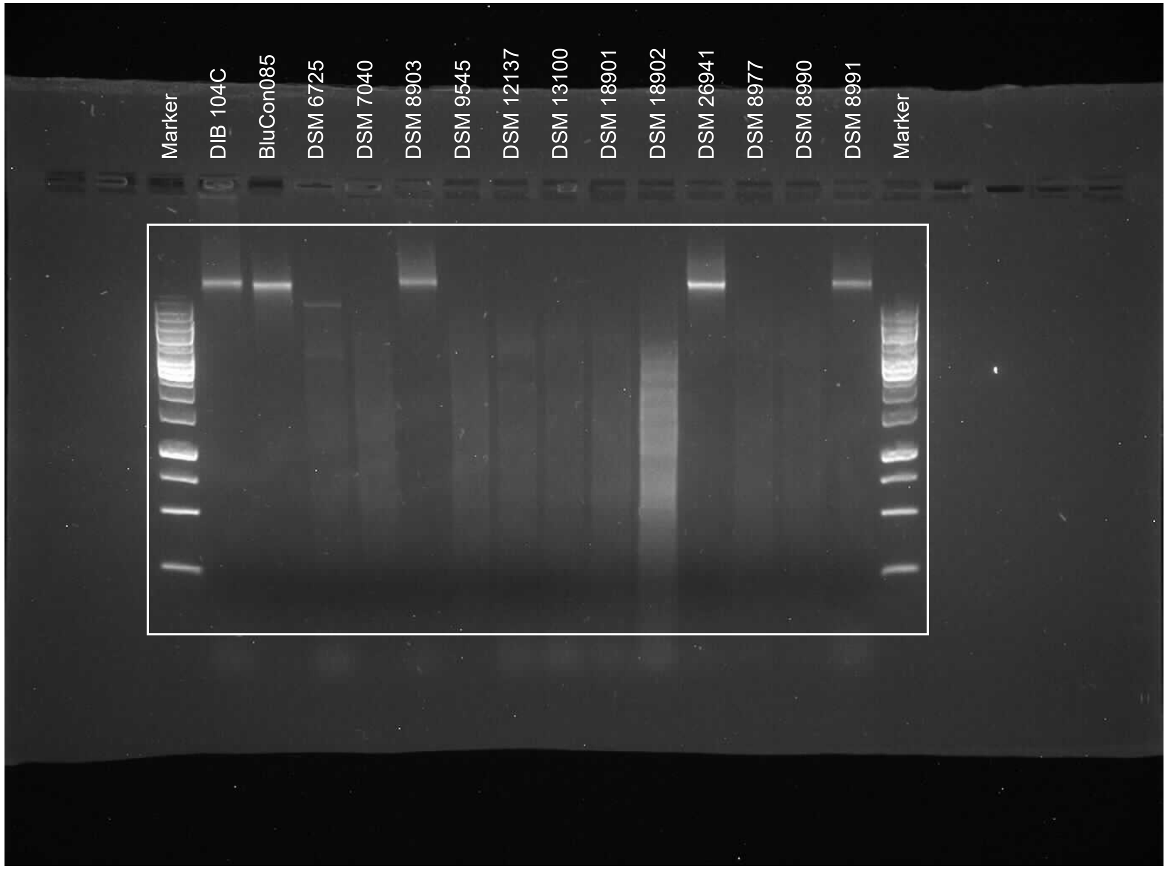

The image was captured with VisionCapt software from Vilber (exposure time of 1.6 sec)

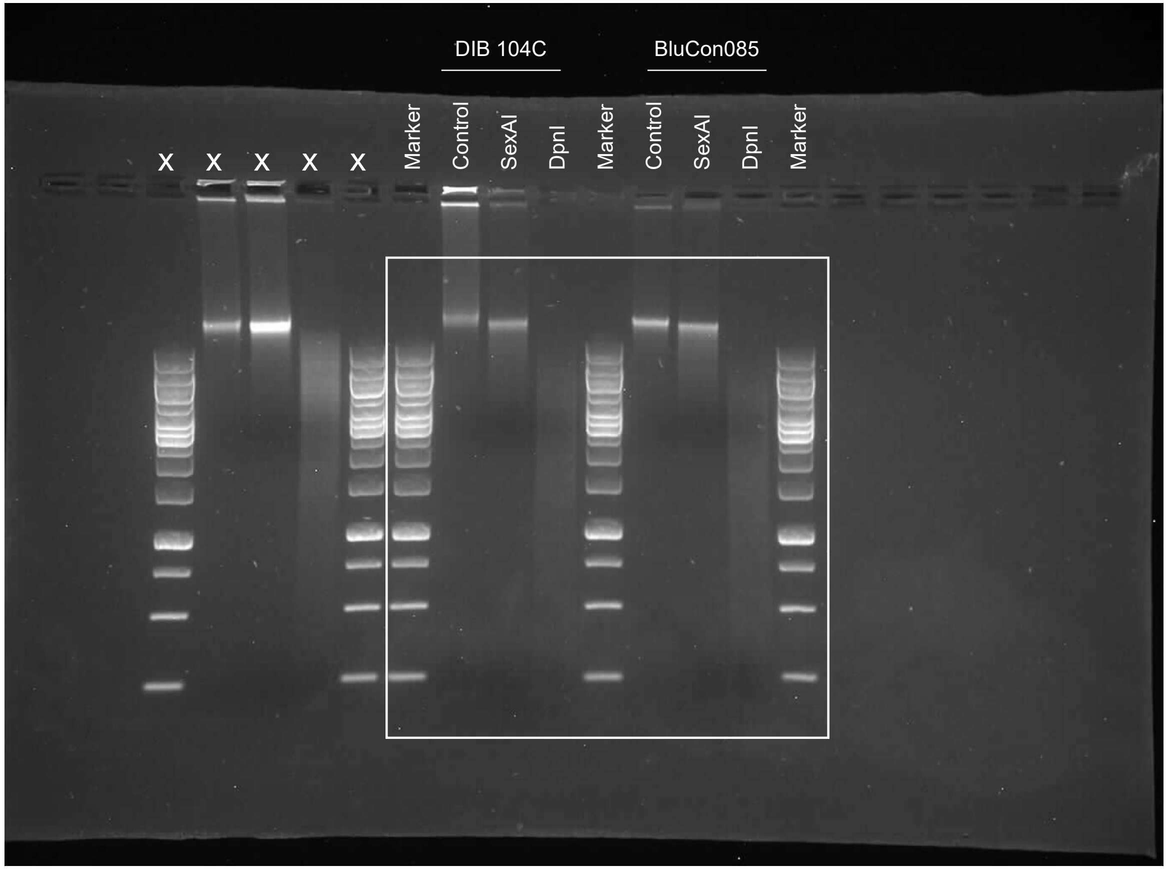

The image was captured with VisionCapt software from Vilber (exposure time of 1.6 sec)
